# Survey on Optimization Methods for LEO-Satellite-Based Networks with Applications in Future Autonomous Transportation

**DOI:** 10.3390/s22041421

**Published:** 2022-02-12

**Authors:** Kaan Çelikbilek, Zainab Saleem, Ruben Morales Ferre, Jaan Praks, Elena Simona Lohan

**Affiliations:** 1 Faculty of Information Technology and Communication Sciences, Tampere University, 33720 Tampere, Finland; ruben.moralesferre@tuni.fi; 2 Department of Radio Science and Engineering, Aalto University, 02150 Espoo, Finland; zainab.saleem@aalto.fi (Z.S.); jaan.praks@aalto.fi (J.P.)

**Keywords:** Low Earth Orbit (LEO) satellite networks, autonomous transportation, optimization criteria, multi-target design, space segment, ground segment, user segment

## Abstract

Future autonomous transportation is one of the most demanding application areas in terms of connectivity, as it has to simultaneously meet stringent criteria that do not typically go hand in hand, such as high throughput, low latency, high coverage/availability, high positioning and sensing accuracies, high security and robustness to interferences, etc. In order to meet the future demands of challenging applications, such as applications relying on autonomous vehicles, terrestrial networks are no longer sufficient and are to be augmented in the future with satellite-based networks. Among the emerging satellite networks, Low Earth Orbit (LEO) networks are able to provide advantages over traditional Medium Earth Orbit (MEO) and Geo-Stationary Earth Orbit (GEO) networks in terms of signal latency, cost, and performance. Nevertheless, several challenges exist in LEO system design, which have not been fully addressed in the existing literature. In particular, the problem of LEO-system optimization of design parameters is a multi-dimensional problem with many aspects to be considered. This paper offers a comprehensive survey of the LEO-system design parameters, of the challenges in LEO system design process, and of the optimization methods for satellite communication, positioning, and sensing applications, as well as a summarizing discussion on the design considerations for LEO-based networks to support future autonomous transportation.

## 1. Introduction

Terrestrial, maritime, and aerospace transportation solutions are increasingly relying on automated tasks and on energy-saving enhancements, such as route optimization, edge and cloud processing via machine learning algorithms, as well as the convergence of communication, positioning, and sensing tasks at the receiver side or in the edge/cloud. The networks needed to support the wireless tasks in future transportation solutions are no longer limited to terrestrial networks, but they are being expanded with satellite-communication networks, such as those based on LEO satellites. Such expansion is needed in order to increase the coverage areas and the end-user ubiquitous access to wireless services and to provide equal accessibility worldwide. LEO orbits have altitudes ranging between about 200km to 2000km above the Earth’s surface (below the Van-Allen radiation belts), which makes LEO satellites cheaper to build and launch in comparison with satellites launched to MEO and GEO orbits. The lower costs of building and launching LEO satellites (compared to MEO and GEO ones) have also enabled better commercial viability of autonomous-transportation services. As a result, there is a significant effort worldwide to build new LEO-based systems for a variety of broadband and narrowband communications (e.g., Iridium, Oneweb, Starlink, Kuiper), Internet of Things (IoT) solutions (e.g., Hiber, Astrocast, Athena, Myriota) Earth Observation (e.g., Iceye, RapidEye, Capella Space), autonomous transportation (e.g., Pulsar, GeeSpace), and, possibly, new Position, Navigation, and Timing (PNT) systems. Integrative solutions of edge/cloud solutions with LEO have already been proposed, e.g., in [1].

Many LEO communication mega-constellations are already deployed in the sky, such as SpaceX Starlink, OneWeb, Amazon Kuiper, accompanied by smaller-sized more specialized constellations, for example, for IoT applications, such as Myriota, Hiber, Inmarsat, and others. In addition, the emerging concept of Low Earth Orbit-based Positioning, Navigation, and Timing (LEO-PNT) [2,3,4,5] is receiving more attention in the research world by focusing on alternative satellite-based navigation methods via LEO satellites.

Based on payload applications, LEO constellations can be broadly distributed into three categories: (i) remote sensing (which includes Earth Observation); (ii) wideband and narrowband communications; and (iii) navigation—with the latter having extensive use in transportation and logistics. In terms of orbital altitudes, LEO orbits present practical advantages over MEO-based solutions in terms of lower-latency communications, shorter positioning time, possible higher positioning accuracy, higher image resolution and lower launching, building, and maintenance costs than MEO and GEO satellites [6].

A novel challenge brought in by the combination of intelligent transportation solutions with LEO-based wireless links is the high-speed relative motion between the LEO satellites and the vehicle of interest, which can be in the order of thousands of meters per second. The design of future LEO systems should be able to take into account not only the target application scenario (such as intelligent transportation via high-speed trains or Unmanned Autonomous Vehicle (UAV)) but also the multi-dimensional services to be offered by future LEO systems in terms of communication, positioning, and sensing targets. Optimization methods to ensure such co-design are tremendously important and have not yet been addressed in the context of designing LEO-system parameters to the best of the Authors’ knowledge. Two LEO systems are currently being built with the specific target of future mobility and unmanned vehicles, namely GeeSpace from the Geely Technology Group in China and Pulsar from Xona Space Systems in the United States. Currently, there is very little public information about the design parameters of these two systems. We will include a discussion about the known design parameters of these systems later in our paper.

The aim of this paper is to offer a comprehensive survey of optimization methods that are showing promising results and can be used in the context of LEO-system design and also to provide examples and design recommendations for chosen scenarios, covering both the LEO space components (i.e., constellation optimization) and the Earth and vicinity-to-Earth components (i.e., ground segment and receiver optimization for terrestrial and aerial vehicles). The chosen application area is the area of the intelligent transportation systems, as this is a broad-encompassing area covering multi-mode receivers (terrestrial, maritime, airborne), all-speed scenarios (from stationary receivers to ultra high-speed receivers), and challenging constraints in terms of communication, positioning, and sensing target metrics. To sum up, this paper’s contributions are:

- An overview of LEO system design considerations for various applications, including high-speed intelligent transportation;
- A comprehensive survey of optimization methods for LEO system design, targeting challenging application scenarios, such as future autonomous transportation;
- The target optimization metrics and typical optimization problems involved in the three-segment architecture of any LEO system presented in a compact form;
- Addressing in detail the space segment and constellation optimization by taking into account aspects not widely addressed so far in the current literature, such as launch and maintenance costs and payloads, constellation management and scaling, and topology models;
- Summarizing, in a concise form, **the optimization trade-offs** related to space, ground, and user segments in LEO design, targeting the performance metrics specific to all-speed (and in particular to high-speed) scenarios of autonomous vehicles;
- Design recommendations for future LEO systems for navigation, sensing, and communication purposes.

The remainder of this paper is structured as follows: Section 2 covers an overview of the related works from the literature that either have a similar purpose to our paper or showcase the general status of the LEO networks. Section 3 introduces the general architecture of LEO systems and the different segments where optimization can be utilized. Section 4 shows the mathematical formulation of generic optimization problems and classifies the optimization methods. Section 5 introduces high-speed scenarios that arise within the LEO networks and explains the requirements of such systems. Section 6 details the optimization problems of interest within the space segment and provides examples of optimization objectives, criteria, parameters, and methods. Section 7 and Section 8 repeat this process for ground and user segments. A few illustrative simulation-based examples are also included here. Last but not least, we provide our recommendations for selecting optimization tools for LEO networks in Section 9 before finalizing the paper in Section 10.

## 2. Related Works

The work related to LEO system design and optimization for autonomous transportation applications is typically focused on only one of these two domains: a LEO focus only or a focus on the intelligent/autonomous transportation side only. Moreover, the papers with LEO tend to only focus on one LEO segment at a time, among the three architectural segments (space, ground, and user), which are described in more detail in Section 3.

Few papers are also addressing the LEO and autonomous transportation aspects. For example, the authors in [7] focused on LEO networks for communication between autonomous vehicles, with a testbed example based on a UAV. No optimization aspects were addressed and the speed of the UAV used in the testing was not mentioned. The two LEO commercial systems targeting the automotive industry, namely Pulsar of Xona Space [8] and GeeSpace of Geely Technology Group [9], have very little public-domain information regarding the design parameters or the adopted optimization steps. It is known that both Pulsar and GeeSpace systems aim at offering centimeter-level positioning to end users and acting as enhancers of Global Navigation Satellite Systems (GNSS)-based positioning technology, but the exact design parameters and mechanisms for achieving these targets are not yet available in the open literature.

The authors in [10] focused on ground-segment optimization of large LEO constellations. The optimization metric was the overall system capacity and the optimization output was the number of ground stations. Monte-Carlo (MC) optimization was employed.

Similarly in [11], the authors presented their review of the literature surrounding marine systems and unmanned vehicles, with numerous real-life and academic examples of state-of-art systems, which included high-speed scenarios that utilized LEO satellite systems, such as Iridium. Particularly, the work in [11] considered LEO networks in terms of remote-control applications. However, the focus mostly stayed on the transportation domain; the satellite constellations themselves were only briefly mentioned and optimization aspects were not discussed for any scenario.

The work in [12] addressed the problem of navigation services via MEO and LEO satellites for autonomous vehicle applications, which have stringent positioning requirements of decimeter-level accuracy. Several possible LEO advantages in terms of complementary positioning methods to MEO GNSS were listed, such as stronger received signals, better resilience to interference, and fast LEO -satellite speeds, enabling carrier phase differential precise positioning. No optimization method was discussed in [12], but several optimization metrics were presented, such as Carrier-to-Noise Ratio ($C/N_{0}$), jamming mitigation ability, and material-penetration ability (e.g., LEO signal penetration through brick or concrete walls).

In [13], a Genetic Algorithm (GA)-based optimization, with Geometric Dilution of Precision (GDOP) and the number of satellites as optimization metrics, was employed for space-segment optimization of a LEO-based navigation system relying on a Walker constellation. It was found that good LEO coverage for navigation purposes can be reached with constellations between 180 and 264 and satellites placed at orbital altitudes between 900 and 1500 km.

The work in [14] explored possible Dense Small Satellite Network (DSSN) applications on LEO networks, focusing mostly on the DSSN in terms of its architecture, requirements, and performance. The work did a good job in determining parameters that needed to be optimized for DSSN networks and determined the boundaries the LEO networks were subject to, but it did not address the actual process of optimization. However, some optimization methods for resource management were mentioned but not presented in detail.

In [15], the authors proposed a Software-Defined Networking (SDN)-enabled LEO constellation satellite network and formulated an optimization problem for SDN controller placement and assignment. They also developed mathematical models and provided SDN network-related cost metrics, such as migration and reconfiguration costs. While the study covered SDN network optimization in great detail, it limited its scope by focusing the analysis on the Iridium constellation, effectively excluding optimization aspects regarding the constellation itself.

The work conducted in [16] analyzed the handover performance in a Low Earth Orbit-based Non-Terrestrial Network (LEO-NTN) via system-level simulations, with a focus on the ground-segment optimization. The work was separated into two phases. First, it conducted a performance analysis of a conventional Fifth-Generation of Cellular Networks (5G) new radio handover algorithm in LEO-NTN scenarios in order to find optimal parameters with respect to chosen key performance indicators. Secondly, there was a comparison in several terrestrial scenarios based on urban macro-scenarios with high-speed trains.The results in [16] showed that the mobility results were dominated by handovers happening too late, which were causing failure cases. However, due to the assumptions made within the simulations, the study in [16] focused solely on the ground segment, and it did not take into account independent or joint optimizations aspects of the space and user segments.

The authors in [17] considered a Speech Emotion Recognition (SER) application for autonomous vehicles, for which a 5G-enabled Space-Air-Ground Integrated Network (SAGIN) was designed. As such, the work explored ground, space, and user segments for the application of interest, but it only provided the design and architecture of the network, without explaining the related optimization processes. However, the user-segment optimization related to SER was covered in great detail, as they explained the Artificial Intelligence (AI) model used for acoustic data modeling.

The authors in [18] discussed LEO communication architectures to support high-speed UAV, and, in particular, the resource allocation in uplink connectivity. A convex optimization problem in terms of throughputs and energy-efficiency metrics was formulated in [18] and solved via Matlab CVX software (a software for disciplined convex programming).

Table 1 summarizes the related work and what we bring with respect to existing studies and surveys.

## 3. The Three-Segment Optimization Architecture in LEO

A typical LEO satellite network has a three-segment architecture:

- **Ground Segment** includes the Ground-Station (GS) infrastructure, which serves as a control unit for the satellite constellation and manages internal parameters;
- **Space Segment** includes the satellite constellation (as well as the propagating signals from satellite to ground);
- **User Segment** refers to any and all applications that the system serves (i.e., cellular networks, PNT applications, transportation, UAV etc.) as well as to any LEO receiver.

**Figure 1:**
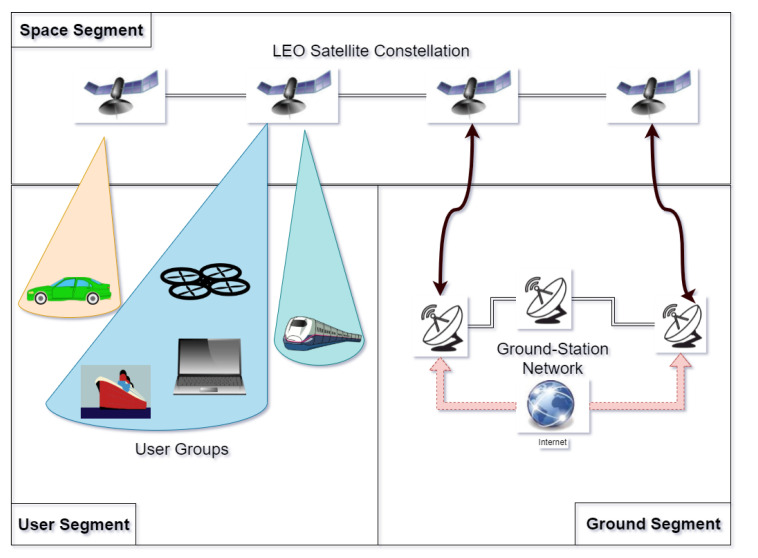
Typical LEO Network Three-Segment Architecture.

Figure 1 provides the ’big picture’ regarding this architecture. The space segment is comprised of satellites in the sky. In LEO constellations, these satellites can carry omnidirectional or directional (beamforming) antennas. The latter case is the one most encountered in LEO mega-constellations nowadays, and it is the one illustrated in Figure 1, where each satellite beam can serve a certain end user. The ground segment hosts the GS satellite network and is complemented by a number of GSs, placed all over the Earth, with the main tasks of monitoring, managing, and controlling the platforms and the signals sent by the satellites. The ground segment typically does not interact with the user segment, but only with the space segment. Last but not least, the user segment comprises all user devices enabled with a LEO-supporting chipset; such devices can serve a myriad of applications needing communication, navigation, and/or sensing capabilities. On-board LEO chipsets on such user devices can also support integration with other chipsets, such as 5G chipsets, IoT chipsets, or Inertial Navigation Sensors (INS). In the case of Earth Observation constellations, the GS network is used mostly for sensor-data downloads. In this paper, we assume this three-segment architecture applies to all LEO networks.

Each of the segments has processes that require optimization; Table 2 summarizes the optimization-related problems that have been actively addressed in the scientific community in recent years for each segment of the above-mentioned architecture. These optimization problems are shown together with examples of optimization objectives, parameters of interest, as well as common metrics used in the optimization process. An important note is that, while the optimization objective of each problem varies, any optimization objective can be categorized according to the target problem, e.g., as shown in Table 2.

Below, we group the optimization criteria related to LEO networks under three main optimization classes. We show some examples for each category, and we specify if the example cost functions are to be maximized (max.) or minimized (min.):

1. **Coverage-related aspects:**
  - Min. Satellite Revisit Time: This revisit time is the time elapsed between consecutive observations of the same point on Earth by a satellite. The lower this time, the better the performance.
  - Max. Satellite Availability: The availability refers to the percentage of time that the service performance provided by the satellite reaches the user equipment in a desired location. Therefore, the higher the availability, the better the system performance.
  - Min. Satellite Orbit Drift: Deviation of the satellite from the planned orbit due to atmospheric drag and gravity.
2. **Cost-related aspects:**
  - Min. Production Cost: This refers to the production and maintenance cost of satellites, GS, and tools; the lower, the better.
  - Min. Launch Cost: This is the cost related to launching satellites to the desired orbits; the lower, the better.
  - Min. De-orbiting Cost: This is the cost to de-orbit a satellite (i.e., take satellite out of the constellation) after its lifespan ended; the lower, the better.
  - Max. Satellite Lifespan: This is the time a satellite spends operating in acceptable conditions; the higher, the better.
3. **Performance-related aspects:**
  - Min. Latency: The time delay before a full data transfer takes place for a communication, sensing, or navigation task. The lower the time, the better the performance.
  - Max. Stability: The property that is inversely related to the need for change within the system. The higher the stability, the better the performance.
  - Max. Throughput: The amount of data (signals, supported number of users, etc.) passing through the system; it is of particular importance for communication-related applications, and typically, the higher, the better. However, some navigation and sensing applications do not require high throughputs; in such cases, throughputs targets may be removed from the optimization parameters.
  - Max. Signal-to-Noise Ratio (SNR) or $C/N_{0}$: SNR and $C/N_{0}$ are measures of the signal quality after unwanted modifications that the signal may suffer during transmission, capture, storage, conversion, and processing. The higher the SNR and $C/N_{0}$, the better; a minimum value for SNR or $C/N_{0}$ typically needs to be guaranteed for good functioning of the system.

While Table 2 cannot cover every possible problem related to the LEO segments, it provides a very good example of both the scope and the complexity of the entire system’s optimization. This paper focuses on the example problems listed Table 2 and on various aspects related to those problems, with the note that additional optimization problems such as those related to packet routing and medium access control may exist, but they fall outside the scope of the current paper.

**Figure 2:**
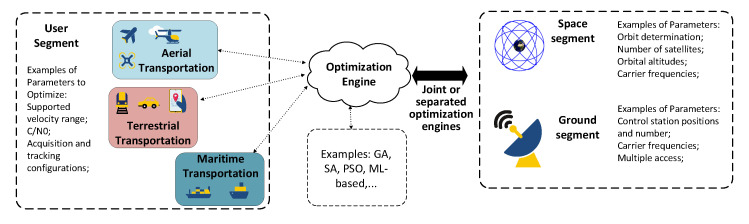
Block Diagram of the Optimization Aspects with respect to the Three-Segment Architecture of LEO-Based Networks.

Figure 2 provides a map for the optimization processes with respect to each of the three architectural segments, with a focus on autonomous transportation regarding the user segment. The main take-away idea from Figure 2 is that certain optimization methods can be used to solve different problems in different segments, as a method’s applicability is determined by problem formulation as well as the problem’s nature.

Although space-segment optimization may deal with additional problems such as controller placement for LEO-based SDN (a controller in terms of SDN is an application that manages flow control for improved network management and application performance), its primary design problem is **constellation optimization**. Constellation optimization is one of, if not the most, critical aspect of LEO space-segment design, as the constellation parameters are directly related with critical operating parameters of all end-user applications, such as communication and navigation on autonomous vehicles. Some examples are: the orbital altitudes directly affect the latency of LEO satellite networks; the orbital plane positioning of satellites directly determine the coverage areas, which, at its turns, is related to the feasibility of user applications. In addition, the optimization of such parameters will have to deal with and successfully satisfy any regulatory criterion set by entities, such as International Telecommunication Union (ITU), Federal Communications Commission (FCC), or any other local or international regulatory entity. Related to the regulatory aspects, physical-layer parameters (e.g., frequency allocations, used modulations, maximum transmitted powers, etc.) must be taken into account in order to keep unintentional interference with the rest of systems (radio astronomy, already functioning GNSS systems etc.) to a minimum degree [43,44]. Additionally, it should not be a surprise that some of the optimization parameters regarding different architectural segments (as well as some metrics) relate with each other in varying proportions, as seen from Table 2.

In contrast to the space segment, the user segment deals with optimization problems that occur for particular application interests, and, as a result, it includes a very large number of optimization-related problems that are generally case-specific. Technically, any application that uses both LEO satellite systems and can be optimized is included in the ’user-segment’ terminology. A simple example would be a communication network with swarm drones that uses LEO satellites, which have optimization aspects ranging from data transmission to network topology to the actual goal of the swarm application, such as obstacle avoidance or drone tracking. Another straightforward example is the satellite-selection problem in GNSS; this refers to finding the optimal number of satellites to track if there are more available satellites than necessary. As GNSS information can used in a variety applications, it is a broad enough problem to be provided as an example in Table 2. Additionally, the satellite-selection optimization problem is also an optimization problem that has to happen at the user-segment side, as it heavily depends on the user/vehicle’s location and continuous motion. Of course, one can only select among visible satellites in cases where coverage is not an issue, and this is also related to the space-segment design. Therefore, the satellite-selection optimization problem is a good example of how problems from different LEO segments relate with one another.

Unlike the other segments, the ground segment is quite straightforward to design, as it is generally the segment that monitors the network for control-related purposes. Due to this aspect, the ground segment has only one significant optimization problem: GS planning. This problem deals with the sky coverage of the GS and focuses on geographic and cost-related aspects.

There also exists optimization problems that require being addressed in all three segments (not present in Table 2) for clarity purposes and because they fall outside the scope of this paper). These include more general aspects of the physical layers specific to different segments, such as the signal and antenna/beamforming-related optimizations, resource-management for individual devices within the network, such as satellites or vehicles, channel coding aspects, and multiple-access optimization. Likewise, some problems require handling from multiple segments, such as channel-based optimization, Multiple Access Channel (MAC) design, handovers, and security. More details about the segment-by-segment optimization problems are presented in the next sections.

## 4. Categorization of Optimization Methods

Before going into high-speed scenarios and examining optimization processes in different segments, it is useful to introduce what constitutes an optimization problem and to provide a classification of different optimization methods based on their optimization objectives.

A typical unconstrained optimization problem can be written as in Table 3, according to one of the four types listed there. These four types are based on whether the inputs and outputs are scalars or vectors, respectively.

The target optimization function f(·) or functions $f_{i}\left(\cdot \right),i=1,\ldots ,N$ can be either minimized or maximized; for simplicity, we formulate everything in terms of a minimization problem, with the equivalence $max_{x}f\left(x\right)=min_{x}\left(-f\left(x\right)\right)$. In Table 3, S is the scalar optimization search space, and $S^{M}$ is the vector optimization search space. The constrained optimization problems can be easily formulated starting from the formulas in Table 3 by adding some constraints or boundaries, such as, for example, $g_{i}\left(x\right)\leq 0,i=1,\ldots ,M_{1}$ or $h_{j}\left(x\right)\leq 0,j=1,\ldots ,M_{2}$, where $g_{i}\left(\cdot \right)$ and $h_{j}\left(\cdot \right)$ are functions defining the constraint on the solution *x*.

When we have a single-objective or a multi-modal optimization problem (i.e., a single scalar function of a scalar or vector input), there is usually, at most, one optimization solution $x^{*}$ (scalar) or $x^{*}$ (vector).

When we have a multi-objective optimization problem, several objectives ($f_{i},i=1\ldots ,N$) must be minimized simultaneously, and therefore, there usually exists a trade-off between the different functions $f_{i}$. The optimal solution is called *Pareto optimal* $x^{*}$ (scalar) or $x^{*}$ (vector), and it is a set of non-inferior solutions in the sense that no other solutions can be found that can minimize one of the objective functions $f_{i}$ without increasing the value of another of the objective functions $f_{j},j\neq i$.

**Figure 3:**
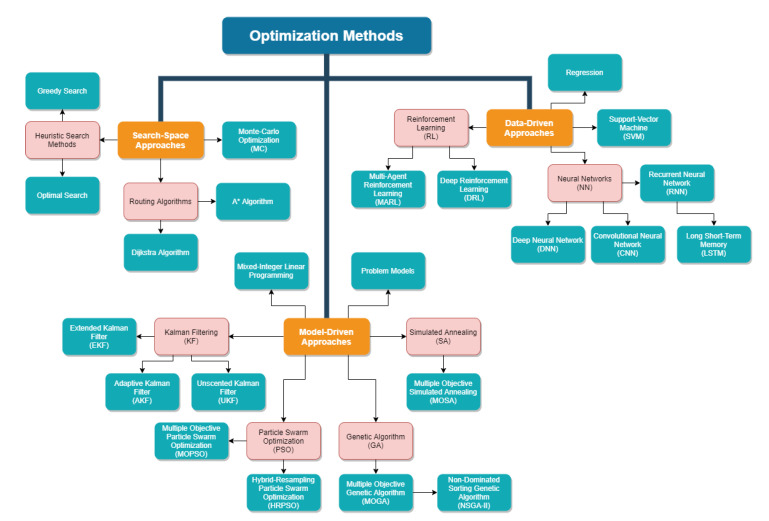
Categorization of Optimization Methods.

Furthermore, we classify the optimization methods that have applications in LEO networks according to Figure 3. It is straightforward to see the relation between Figure 3 and Table 2, as Figure 3 includes most of the methods listed in Table 2. However, it is also important to show the relation between Table 3 and Figure 3 by noting which methods are suitable for which types of optimization problems. We will focus on explaining this relation for the remainder of this section.

On one hand, many of the methods in Figure 3 are methods inherently developed to deal with single-objective optimization problems in mind; for example, traditional heuristic algorithms, such as the Greedy Search [45] and Dijkstra Algorithm [46], minimize the defined cost (i.e., node or transmitter-receiver distance, network traffic/throughput, etc.). Similarly, linear solvers, such as Mixed-Integer Linear Programming (MILP), are also applicable for single-objective optimization problems; however, they can also be expanded to operate with multi-objective or multi-modal problems, given accurate and cleverly formalized problem models, e.g., as shown in [47].

On the other hand, recent and advanced methods are suitable for all types of mathematical optimization problems seen in Table 3. The complex search algorithms, such as Simulated Annealing (SA) [48], and evolutionary algorithms, such as GA [49] and Particle Swarm Optimization (PSO) [50], are methods that originally dealt with a single-objective optimization problem again, but they now have improved versions that extend the method to multi-modal and multi-objective problems (i.e., Multiple-Objective Simulated Annealing (MOSA) [51], Multiple-Objective Genetic Algorithm (MOGA) [52], Non-Dominated Sorting Genetic Algorithm II (NSGA-II) [53], Multiple-Objective Particle Swarm Optimization (MOPSO) [54], and Hybrid-Resampling Particle Swarm Optimization (HRPSO) [55]). Similarly, the Neural Network (NN)-based methods [56], such as Deep Neural Network (DNN) [57] and Convolutional Neural Network (CNN) [58], are typically designed for a particular problem, which can be any type of optimization problem from Table 3.

Other methods, such as Kalman Filtering (KF)-based methods (i.e., basic KF [59], Extended Kalman Filter (EKF) [60], Unscented Kalman Filter (UKF) [61], Adaptive Kalman Filter (AKF) [62]), and Support-Vector Machine (SVM) [63], are methods that solve single-objective multi-modal optimization problems, but, similarly to the above-mentioned cases, they are flexible in the sense that they can be extended to other types of optimization problems via modifications. KF methods, in particular, are very suitable for estimation problems under accurate underlying problem models.

An important method to discuss in detail is the MC optimization. Despite also being a brute-force optimization method that relies on the problem model, MC is unique in the sense that most simulations regarding LEO networks are constructed as a Monte-Carlo loop due to its ability to directly propagate parameters through the system model. This makes it so that the methods that require environment observations or interactions, such as evolutionary algorithms, are methods that are jointly utilized with MC loops. However, it should also be noted that the MC loop does not refer to an MC optimization in such cases; the parameters are optimized according to the actual optimization method utilized. For detailed explanations regarding MC, we advise dedicated sources such as [64,65].

Since LEO networks have a high number of optimization-related problems from all the types shown in Table 3, it should not be surprising that many of the discussed methods are suitable for multiple types of optimization problems. However, it should be noted that a method being suitable for a problem does not mean that it performs optimally or that it is feasible to apply for that particular problem. We will discuss the differences and trade-offs between different methods in detail in the next sections, when we examine the optimization problems in different segments of a LEO system.

Last but not least, an important aspect, which has a significant impact to the overall system in practical applications concerning autonomous vehicles, is the overhead of the optimization engines. In the case of an online optimization (i.e., optimization that takes place during the system operation), the overhead mainly refers to the time delay added to the system by the optimization procedure as well as to the required system resource allocation to perform the optimization. Examples of online optimization tasks are network routing and handover management (see Table 2).

When we perform the optimization before the system is operational, this is referred to as an offline optimization part. Examples of offline optimization are the GS planning and constellation optimization (see Table 2). In an offline optimization task, the main overhead is the required time-to-converge and complexity of the optimization method. These two parameters typically determine the feasibility of a method.

The variety of the implementation specifics, such as the chosen implementation language (e.g., Python, C, C++, etc.), as well as the available hardware will have an impact on the system overhead. We will provide additional observations with respect to the overheads when we present the optimization methods in detail in later sections. Generally, the overhead of an optimization method needs to be examined on an implementation-and-scenario-dependent basis.

## 5. High-Speed Scenarios Based on LEO Satellites for Future Autonomous Vehicles

An autonomous vehicle, or a driver-less vehicle, is a vehicle that operates itself and performs necessary functions with minimal (or no) human intervention through its ability to sense its surroundings. There are six levels of automation according to [66]; level 0 refers to the no automation case where the vehicle is completely dependent on a human driver and level 5 refers to the full-automation case, where the vehicle is completely independent in all cases and necessitates no human intervention. Most of the realistic autonomous vehicles nowadays are between levels 2 and 4.

For autonomous vehicles, the notion of high-speed scenarios include any case that deals with high speeds regardless of their domain; a high-speed case can be as simple as providing cellular connectivity to mobile devices inside a fast-moving train, going over 250km/h, or it can be as complex as the need of instant wireless communication between different high-speed drones and flying taxis to avoid collision. For our discussions in this paper, we will consider LEO-satellite system scenarios that either require high speeds in parameters, as in the drone example, or scenarios that occur in high-speed vehicle motions, as in the train example.

If we aim to achieve full automation in high-speed cases, future networks of autonomous vehicles will have stringent requirements in terms of communications, positioning, and sensing characteristics, which are not yet fully met by current cellular and IoT technologies. A summary of these stringent requirements is given in Table 4, together with example studies from the literature that have addressed these challenges to some extent and offered various solutions to them. It is also straightforward to see that the requirements listed in Table 4 can be perceived as boundaries for the optimization problems that have the overlapping metrics from Table 2.

However, it is also important to discuss existing limitations of high-speed scenarios for autonomous vehicles. One limitation in the current terrestrial technologies in terms of autonomous vehicle services is the requirement for ubiquitous and seamless coverage, which should be as close to 100% as possible. In order to address this challenging limit, satellite-based networks, such as leo, as well as their integration with terrestrial networks, have begun being investigated in the literature, e.g., in [78].

Another very important aspect, which becomes even more significant in high-speed scenarios due to time/delays and computation constraints, is the security aspect. Good and up-to-date surveys on the security aspects in automotive transportation and unmanned vehicles can be found in the literature, for example, in [79] (blockchain solutions for UAV), ref. [80] (physical layer security for UAV), ref. [81] (quantum cryptography for UAV), ref. [82] (integrated network security for terrestrial and aerial transportation), ref. [83] (a systematic literature review on AI in UAV safety), etc. Security aspects are very broad and are not typically a part of the design optimization of the space or ground segments, as there are many security solutions that can be devised in the post-design stage, such as using multi-system multi-frequency receivers, using various encryption methods or authentication signals, etc. While we highly recognize the importance of ensuring high security mechanisms for communication, sensing, and positioning purposes in autonomous transportation, it is our opinion that we cannot deal with the security aspects as normal optimization parameters, and therefore, the security part is seen as outside the scope of the current survey. That being said, we would like to point out that some of the optimization methods that we cover in this paper are also utilized in security-related aspects in autonomous vehicle implementations. Some examples include: [84], where the authors implement Reinforcement Learning (RL) to maximize the robustness of UAV dynamics control to cyber-physical attacks, and [85], where the authors implement a Proportional-Integral Derivative (PID) controller using PSO as a tuning method to achieve high stability.

Furthermore, another optimization aspect that directly relates to security is resource management. Generally speaking, security measures require their own share of computational resources, so any optimization that handles management of resources within a LEO system must consider the requirements of the possible security applications, even if as briefly as only a threshold or reserve. Such threshold is typically straightforward to define as an additional boundary in the optimization problem.

A similar, scenario-based limitation that arises frequently in LEO satellite systems is the problem of the satellite handovers (i.e., changing end-user connection from one satellite to another), which heavily impacts the requirements of autonomous vehicle systems in terms of latency, throughput, and accuracy. Due to the low orbital altitude ( 200–2000 km) of LEO constellations, LEO satellites move at higher speeds (i.e., 7.5 km/s at 600 km altitude) than MEO and GEO satellites [16]. This effectively means that, for any particular autonomous vehicle, the satellite is available for only up to a few minutes before a handover is required [14], even when the LEO constellation is dense enough to provide 100% coverage for the area of interest. The frequency at which the handovers happen changes dynamically as the vehicle moves, and it can highly affect the latency, throughput, and accuracy targets, especially in high-speed motion scenarios with opposite directions with respect to the satellite’s movement. Again, this remains a limitation that is being investigated to various degrees by academia in studies such as [40,42].

## 6. Space-Segment Optimization Aspects

As mentioned earlier in Section 3, the main optimization problem of the space segment in LEO design is the constellation optimization. This section focuses on LEO constellation characteristics and constraints through a detailed survey of significant design elements, existing constellations, and key performance parameters, as well as applicable optimization methods.

The main goal is to show the steps to adopt toward a feasible and optimized constellation design to provide service to transportation vehicles and to meet the emerging demands of more autonomy and reliability.

There are many approaches in constellation optimization and one area that is still lacking in the current literature is the area of multi-target and restriction-driven optimization. Pure geometry-based solutions are easy to sketch and small satellite constellations are not necessarily demanding in terms of the minimum number of satellites. However, a realistic optimization of the constellation should take into account the factors that are most restrictive, such as the orbit topology, satellite launch cost, radiation requirements [86], satellite redundancy, atmospheric drag (i.e., the force exerted on satellites due to their movement through air), the need for propellant and maneuvering, etc. All these aspects are addressed in detail in the following subsections.

### 6.1. Orbit and Constellation Elements

The selection of the orbital parameters for the constellation affects the overall design and mission output. In Table 5, the main parameters describing a constellation are depicted, which can be leveraged to optimize the constellation [87].

The orbital parameters of the constellation generates its topology mainly dominated by the type and region of coverage. The coverage can be regional, zonal, or global with continuous or intermittent visibility. The coverage is typically 1-fold to 4-fold. The communication and surveillance applications usually require 1-fold coverage, and 4-fold coverage is essential for positioning and navigation applications [92].

**Figure 4:**
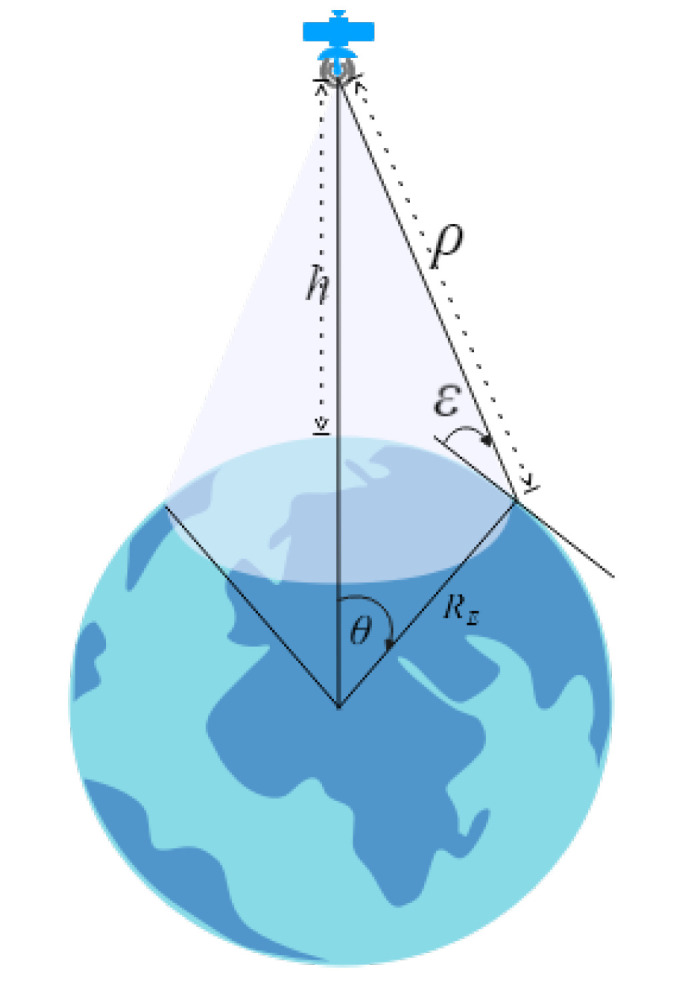
Satellite Coverage.

The coverage equation is modeled by

(1) $$C=\cos\left(\theta +\varepsilon \right)=\cos\left(\frac{\varepsilon }{1+\frac{h}{R_{E}}}\right)$$

where C is the coverage parameter, ε is the elevation angle of the viewing cone of the satellite, *h* is the satellite altitude, $R_{E}$ is the Earth radius, and θ is the central angle of coverage [87]. An illustrative example of these parameters is provided in Figure 4.

### 6.2. Constellation Topology

The major constellation topologies are the following four topologies:

- **Street of Coverage Constellations:** Multiple satellites placed in circular or near circular orbital planes with the same altitude and inclination and phase separation creates a Street of Coverage constellation. The number of orbital planes (streets) then determine a zonal or global coverage and can be optimized accordingly. The planes are distributed in a non-symmetric way [87,93].
- **Walker Constellations:** A Walker constellation contains satellites in circular orbital planes with the same altitude and inclination distributed symmetrically along the equatorial reference. The inclination depends on coverage over the area of interest on the globe. A Walker constellation includes the star pattern, where orbital planes are evenly distributed over 180 degrees of the Right Ascension of the Ascending Node (RAAN) range, and the delta pattern, where planes are distributed over 360 degrees RAAN range. The rosette pattern is similar to delta based on Ballard’s earlier work. The other constellations based on Walker Delta include sigma pattern and omega pattern. An example constellation is the Galileo satellite constellation, which is deployed in a Walker 24/3/1 in MEO, where 24 satellites are distributed in 3 orbital planes inclined at 56° [94]. Another one is the Iridium constellation, which is a 66/6/2 Walker Star constellation with a near polar inclination of 86.4°. The 66 satellites are distributed in 6 orbital planes [95].
- **Draim Constellations:** A Draim constellations use elliptical orbital planes with a similar period and inclination to achieve 1-fold or multi-fold continuous global coverage with fewer satellites than required for constellations with circular orbit or near circular orbits.
- **Flower Constellations:** A Flower constellation is a symmetrical constellation proposed by Mortari et al. [96] in a rotating frame of reference giving flower-like geometry. They have similar values for semi-major axis, eccentricity, inclination, and argument of perigee and differ in mean anomaly and RAAN. The Flower constellations were modified in a 2D lattice flower by Avendaño et al. [97] and 3D lattice flower by Davis et. al [98] models and 2D necklace flower [99] and 3D necklace flower constellation [100]. Walker and flower constellation configurations provide global coverage [101].

Table 6 presents the mathematical modeling of the two most encountered constellation topologies, namely Walker and Flower constellations. In Table 6, *i* is the inclination of orbit, $N_{T}$ is the total number of satellites, $N_{P}$ is the number of orbital-planes of the constellation, *F* is the relative phasing parameter between adjacent orbital planes, and *h* is the Satellite (Sat) altitude. $\Delta \Omega_{jk}$ is the RAAN and $\Delta M_{jk}$ is the mean anomaly with respect to the reference Sat, with *j* as the orbital plane number and *k* as the Sat number within the orbital plane. For Flower constellations, $N_{p}$ is the number of orbital revolutions of Sat, and $N_{d}$ is the number of rotations of the rotating reference frame; for the Earth-centered, Earth-fixed frame (ECEF), $N_{d}$ is equal to the number of days. These design parameters are related as $N_{p}\frac{2\pi }{n}=N_{d}\frac{2\pi }{\omega_{E}}$. Here, *n* is the orbital mean motion of the Sat, and $\omega_{E}$ is the angular velocity of the rotating frame. $F_{d}$, $F_{n}$, and $F_{h}$ are independent integers for phasing. $\Delta \Omega_{k}$ is the RAAN, and $\Delta M_{k}$ is the mean anomaly, with *k* as the Sat number with respect to first Sat [101,102].

**Figure 5:**
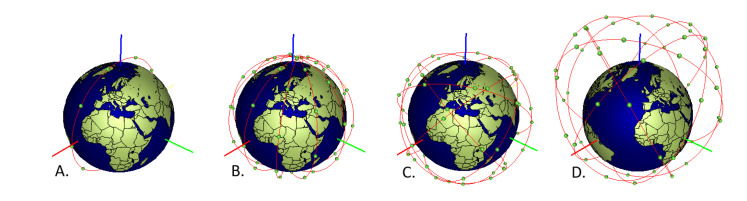
Examples of Constellations: **A**. Single orbital plane with 10 satellites at 700 km. **B**. Star constellation or Walker constellation with 6 polar orbital planes, with 10 satellites at 700 km. **C**. Walker constellation with 6 orbital planes at a 53 degree inclination and 10 satellites in every orbital plane. **D**. Flower constellation with 6 orbital planes at a 63 degree inclination, with 10 satellites on elliptical orbit with apogee of 3000 km.

Figure 5 visualizes examples of constellations at various altitudes and with various number of orbital planes and inclinations.

### 6.3. LEO Satellite Constellations

In the literature, we can find many constellations that have most of their satellites already in the sky (e.g., Globalstar, Orbcomm, Iridium, …). However, not all LEO constellations are fully operational. Some of them are partially deployed (e.g., Starlink, Oneweb), and some others are only planned to be launched in the relatively near future (e.g., Amazon Kuiper or Facebook Athena). In Table 7, we have listed some of the most promising/currently relevant LEO constellations. Currently, LEO constellations are typically used for narrowband and broadband communications and Earth observation. The main advantage of LEO lower altitudes (compared to MEO) is the relatively low latency, fundamental for voice applications and high performance internet connection. Traditionally, LEO constellation were placed in the higher end of LEO altitudes (e.g., >800), e.g., Globalstar, Orbocomm, and Iridium. On the contrary, due to the latest progress in satellite construction and launching, we are able to put in orbit smaller satellites in a more efficient way, placing in an orbit multiple satellites in a single launch. Besides the constellation names, Table 7 also lists some relevant parameters describing these constellations, as well as some references for the most advanced readers. Table 7 summarizes fundamental orbit parameters, such as the total number of satellites in the constellation, the number of independent orbital planes $N_{P}$, the altitude (or altitudes) *h* considered during the constellation design, and the orbital plane inclination *i*. In addition, Table 7 also shows the frequency band used for the considered constellations, as well as the typical satellite mass and the main purpose of the constellation.

### 6.4. Launch Services and Constraints

The new-space approach offers exponential growth in contrast to the traditional space by making use of small satellite technology, new manufacturing techniques, commercial off-the-shelf components, by taking relatively higher risks, and by applying fast development cycles. This has prompted an ecosystem where more and more satellites and constellations are being deployed to offer terrestrial services for emerging markets. With new financing models, the outcome is novel and comprised of futuristic space companies, platforms, subsystems manufacturers, launch providers, and ground station services [115,116,117]. With the new-space ecosystem, the launch services have also emerged in new categories [118,119].

- **Dedicated Launches**: A dedicated launch has one major payload that controls the mission requirements on the whole.
- **Traditional Ride-share Launches**: Standard ride-sharing consists of a primary mission where surplus mass and volume used by other satellite missions.
- **Dedicated Ride-share Launches**: Dedicated ride-share launches are multi-mission launches to deploy dozens of satellites with relatively similar orbital parameters.

To meet the requirements of new-space missions, launch broker and services providers joined the launch vehicle manufacturers with reinvented business practices. The Launch Service Provider (LSP) matches a spacecraft with a launch opportunity, providing a standardized separation system with physical integration of the spacecraft to the launch vehicle and with the management of the launch campaign. This has further lowered the barriers for new and nontraditional players to deliver a product to space.

With traditional ride-shares and dedicated ride-shares, the cost of launch has decreased significantly [120]. However, the action of launching satellites as a secondary payload or as part of a ride-share has various constraints, as the launch schedule and insertion orbit are marked by primary payload or through a compromise among all payloads in the ride-share. This also adds restriction on with propellant volume and pressure of any secondary payload’s satellite design, thus limiting the secondary payload to maneuver to preferred orbital planes [121]. For missions with stringent orbital and design requirements, this may not be a feasible solution and a dedicated launch may be too costly [122]. In the case of constellations, such constraint piggyback launches add complexity to the constellation design and optimization [6]. A rough numerical estimate for the overall costs of a micro-satellite (10–100 kg Sat) for a five-year mission lifetime observed by Liddle et al. [123] is given in the following equations, where *C* refers to cost of the part of the satellite, denoted by its subscript.

(6) $$C_{total}=C_{payload}+C_{platform}+C_{launch}+C_{operation}$$

According to [123], the payload, platform, and launch costs are approximately equal to each other, while the operation cost is approximately half of the payload cost (and also of the launch cost).Thus, with the above-mentioned approximations, one could compute the total cost as:

(7) $$C_{total}\approx \frac{7}{2}C_{launch}$$

To acquire the flexibility offered by a dedicated launch with costs comparable to ride-share launches, there is a demand for dedicated micro-launchers. With more satellite clusters and constellations planned to be launched in coming years, many companies are building these launchers to meet these demands [124]. The target of the launch vehicles is to deliver a payload from 10 to 300 kg to LEO with costs matching to the current ride-share costs [121]. Table 8 gives a classification of Launch Vehicle (LV) according to their payload capacity to LEO.

### 6.5. Constellation Deployment

Deployment for an optimal constellation for continuous global coverage requires multiple satellites separated within an orbital plane and distributed over several orbital planes at desired altitudes and inclinations. Deploying a global-coverage constellation with the existing launch paradigms would require a dedicated launch for each orbital plane. This would result in very high launch costs beyond the budget of usual missions [120,125]. As cost is one of the prime optimization parameters, developing an efficient way to deploy a constellation is essential [126]. This requires addressing the constellation configuration, satellite design, and launch opportunities simultaneously [6]. As presented in [127], constellation deployment may be distributed in direct injection, where Sat is placed by LV, and indirect injection, where either the LV or Sat performs non-planner maneuvers to achieve the final orbit. However, Impulse per unit of Mass (needed to perform a maneuver) (ΔV) for such orbital transfers is very high. An alternative indirect injection approach is to utilize orbital perturbations due to Earth’s oblateness to transfer Sat different orbital planes and populate the constellation. This idea was first patented by King and Beidleman to use natural perturbations to separate the orbital planes with RAAN. The nodal precession due to Earth’s oblateness varies with altitude, inclination, and eccentricity at different rates. The nodal precession using Second-Degree Zonal Harmonic of the Earth’s Gravity Field ($J_{2}$) is given by Equation (8) [128], where ${\dot{\Omega }}_{J_{2}}$ is the rate of change in Ω due to $J_{2}$, $R_{E}$ is radius of Earth, *a* is the semi-major axis, *e* is eccentricity, *i* is the orbital inclination, and *n* is the mean motion of the Sat.

(8) $${\dot{\Omega }}_{J_{2}}=-\frac{3}{2}\frac{R_{E}^{2}}{{\left(a\left(1-e^{2}\right)\right)}^{2}}J_{2}n\cos\left(i\right)$$

The plane separation would first require an in-plane maneuver, which requires less ΔV to change the orbit for a different nodal precession rate and after the drift period required for desired plane separation maneuver back to the mission orbit [121]. For constellation deployment, [129] uses drag to achieve in-plane maneuvering and nodal precession to achieve out-of-plane maneuvering. FORMOSAT-3/COSMIC satellite mission is presented in [130], where six deployed satellites used in-plane thrust maneuvers for orbit raising and utilized differential nodal precession to achieve different orbital planes. A detailed study to deploy a multi-plane constellation using nodal precession is also presented in [131].

Using orbital perturbations for plane separation results in a longer time for full constellation deployment, whereas a dedicated launch induces high mission costs, resulting in a trade-off between deployment time and launch cost [125].

### 6.6. Constellation Maintenance

#### 6.6.1. Orbital Perturbations

The orbital elements under the influence of perturbations result in osculating elements. These perturbations are due to Earth’s oblateness, atmospheric drag, solar radiation pressure, and third body effects. These time-dependent osculating elements change differently for satellites at different points in orbital planes. This results in a relative drift between each satellite changing the constellation pattern and ground coverage over time. The in-plane Sat spacing and plane-to-plane RAAN spacing vary over time, requiring maneuvers for corrections. For LEO Sat, the most significant perturbations are due to Earth oblateness, atmospheric drag, and solar radiation pressure [132].

Earth’s gravitational potential due to non-spherical earth is presented in spherical harmonics. $J_{2}$ has the major effect; the higher order zonal, sectoral, and tesseral harmonics in order of magnitude are three times smaller than $J_{2}$. $J_{2}$ produces secular rates in RAAN Ω, argument of perigee ω, and mean motion *n*. The Argument Of Latitude (AOL) *u*, which is defined as the sum of Ω and true anomaly θ, also experiences a secular change as a result of $J_{2}$. The nodal precession is the rate of change of RAAN, whereas the apsidal precession refers to the precession of the line of nodes on the orbital plane. The atmospheric drag affects the semi-major axis *a* and eccentricity *e* of the orbit. $J_{2}$ or drag does not affect the inclination. The third body effects for LEO satellites are also very small in comparison to $J_{2}$. Deviations in *a*, *e*, and *i* will result in secular drifts in Ω, ω, and *n* [93].

#### 6.6.2. Station Keeping

Station keeping refers to maintaining the satellites in a space box within defined tolerances through either absolute station keeping, where the position is maintained with respect to the central body reference frame, or relative station keeping, where the Sat position is maintained relative to the position of other Sat(s). The initial differences in the Sat orbits and perturbations accumulated over time disrupts the constellation geometry and necessitates station-keeping maneuvers. The perturbations in orbit are short periodic, long periodic, and secular, with each requiring different compensation through either in-track or cross-track orbital maneuvers. The major advantages of absolute station keeping reported in [87] in comparison to relative station keeping are a priori Sat position estimates, more robust control, less propellant requirements, less complexity, and cost. It also highlights that for autonomous station keeping, the absolute station keeping is better as it is implemented with a larger sequence of small ΔV maneuvers, rather than a few small impulsive maneuvers. Sat station keeping can be achieved through low thrust maneuvers or impulsive maneuvers. Both maintenance schemes and propulsion systems are widely studied for LEO constellations.

The authors in [133] study the in-plane station keeping of a Walker constellation, considering Earth’s gravity field and solar radiation pressure, and bringing the satellites to the defined tolerance band for the position by two periodic impulsive maneuvers. The study also gives the ΔV estimates for constellation maintenance. The authors in [134] study the station keeping in constellation as a multi-objective optimization problem with minimum fuel consumption and time constraints. The chosen scheme, in-track and cross-track tolerances, as well as the maintenance schemes set the requirements for the propulsion system of the satellites.

#### 6.6.3. Space Radiation

One of prominent problem for electronics in space is the ionizing radiation that disturbs or destroys semiconductors. The primary sources of radiation in space are: the Galactic cosmic rays, the solar proton events, and the trapped radiation in Earth’s magnetic field. The impact of this radiation on the satellite platform and subsystems can be grouped as follows:

- *Satellite Charging and Internal Charging (SCIC)*: It is the accumulation of charge on the outer surface of the satellite or on the interior surfaces. This causes potential variations between the spacecraft surfaces and the ambient plasma, resulting in Electro-Static Discharges (ESD)-related anomalies.
- *Single Event Effects (SEE)*: Single event effects are caused by the impact of high-energy charged-particle-sensitive electronics of Sat subsystems.
- *Total Ionizing Dose (TID) and Displacement Damage (DD)*: The total ionizing dose refers to the energy produced through the passage of electrons and protons through materials, resulting in degradation.

To withstand the radiation environment, the electronic components should be specially designed and qualified for high-radiation environments (radiation hardening), which makes the components very expensive. Lately, the usage of non-hardened components has been growing to meet the price pressure. The radiation in near-Earth space has been concentrated into so-called radiation belts, starting from an altitude around 1500 km to 2000 km. Therefore, based on the operational altitude and region of the SCIC, the satellite designer can estimate the TID to select components and device radiation shielding and protection [135]. Koons et al. surveyed space anomalies from various databases in [136] and concluded that the largest anomalies recorded were due to ESD and charging. The second largest group was SEE, whereas the surface degradation due to radiation damage, especially for solar arrays, formed the third-most group of recorded anomalies. As the radiation level and its type are dependent on orbital parameters, the optimization of Sat electronics depends on orbit design.

#### 6.6.4. Satellite (Sat) Replacement

A failure of one or more satellites can result in the service deterioration for a constellation providing global coverage. In such a case, having spare satellites and a good replacement strategy are parts of the constellation design in order to make sure that, in the case of a failed or terminated satellite, a replacement is deployed without much delay [89].

Cornara et al. discuss replacement-and-spare strategies in [127,137], dividing them into categories with no-replacement planned, launch-on-demand by ground spares, or on-demand manufacturing, Sat spares in parking orbit and in-orbit spares. In the case of navigation-and-communication satellite constellations, continuous service and reliability are achieved by overpopulating the constellation with one or two extra satellites per orbital plane. In [138], an inventory-management approach for mega-constellation satellite replacement was proposed since traditional spare strategies cannot be applied due to limited scalability. The strategy implements spares as a supply chain with ground facilities as suppliers, satellite parking orbits as warehouses, and in-plane orbital spares as retailers in order to minimize the spare-strategy cost. The service ability and the satellite reliability drive the selection of the strategy for replacement.

#### 6.6.5. End-of-Life (EOL) De-Orbiting

In addition to the maintenance during the mission lifetime, a Sat in a constellation requires an EOL scheme to remove satellites from their orbit upon failure or termination. At the end of life, a Sat is required to fulfill the disposal requirement to re-enter the atmosphere within a 25-year limit. This limitation is implemented in national legislation in order to reduce the accumulation of orbital debris and lower collision risks. Moreover, a dysfunctional satellite in a mega-constellation might pose a threat to the constellation itself via collision risk. The orbital decay depends on the ballistic coefficients of Sat and the solar activity at the EOL time. The ballistic coefficient $B_{C}$ is given as:

(9) $$B_{C}=C_{d}\frac{A}{m}$$

where $C_{d}$ is the coefficient of drag, *m* is the satellite mass, and A is the cross-sectional area. The solar activity is indicated by the solar radio flux at a wavelength of 10.7 cm, also referred to as the solar index. F10.7 changes with the 11-year solar cycle changing the minima and maxima. This parameter is important to estimate the Sat orbital decay as drag correlates with the solar cycle.

If the 25-year limitation cannot be satisfied, the mission designers need to employ alternative disposal strategies. These include:

- **Uncontrolled Reentry,** where the decay time of Sat is decreased by changing the Sat area-to-mass ratio physically. This is the most cost-effective disposal scheme. Sometimes, this scheme may initially require one or more maneuvers to facilitate the decay;
- **Controlled Re-entry,** where orbital maneuver are carried out to induce controlled orbital decay and burn-up in the atmosphere. This would require the Sat to maintain attitude and have a propulsion system for de-orbiting. This would also require the maneuvers to be incorporated into design with EOL fuel budget, resulting in increased platform mass and overall costs;
- **Graveyard Orbit Placement,** where the Sat(s) maneuver to graveyard orbit, defined due to lack of its value for space missions. This is common for GEO and MEO satellites [87,137]. The mission design for a LEO constellation would require incorporating an EOL strategy for Sat.

#### 6.6.6. Space Debris

When designing a satellite constellation, one should also address potential space-debris-related problems. A high number of satellites at a certain orbital altitude increases the risk of those satellites colliding with each other. An orbital satellite collision can generate a cloud of debris, which can render even more satellites nonfunctional and can degrade the space-segment functionality. A collision with satellites belonging to other owners can also lead to liabilities with heavy economic consequences. Even when collisions can be avoided by avoidance maneuvers, frequent maneuvering will increase operational cost and will require in-orbit consumables. Therefore, the collision risk and the required avoidance maneuvers should be estimated for the space segment.

To assess the collision risk, typically, an annual collision probability with a large space object along with orbital lifetime is estimated. The estimation requires an estimate of the satellite projection area, the orbital parameters of the satellite, and the satellite launch date. The collision risk analysis is usually performed using a predictive model database of space objects and space debris. The same database is required for collision avoidance maneuver budget estimation. For example, the European Space Agency (ESA) provides a MASTER (Meteoroid and Space Debris Terrestrial Environment Reference) database and a DRAMA (Debris Risk Assessment and Mitigation Analysis) software package for this task. The space-segment optimization for space debris avoidance is a complex task, which cannot be easily integrated into the general optimization tasks addressed in this paper, and it is omitted in the current framework.

### 6.7. Optimization Metrics Related to the Space Segment

The space-segment optimization, as mentioned above, includes all optimization steps that take place within the satellite constellation, and such optimization steps can happen in multiple layers. An example analogy is the network optimization in [15]. However, in the broadest meaning of the term, the space-segment optimization refers to the parameters of the constellation, see, e.g., Table 5 (and thus named ‘constellation optimization’). The aim of constellation optimization is to distribute multiple satellites with similar types of functions into similar or complementary orbits in order to accomplish specific tasks under shared control.

It is possible to classify the optimization objectives that fall into the chosen application area into the following three categories: (i) coverage, (ii) cost, and (iii) performance (see also the discussion in Section 3). As the space segment is the first LEO segment for which we discuss specifics, we will provide the commonly used metrics following this categorization. For the ground and user segments, our focus will be solely on presenting the metrics rather than where they fit in the categorization.

The coverage metric, which refers to the ground coverage of the satellites from the perspective of the space segment, enables whether or not the target application (e.g., communication, positioning, or sensing) is able to operate in a stable manner. It is traditionally calculated geometrically, and ensuring its continuity is optimized via secondary metrics, such as minimizing the satellite revisit time or maximizing daily visibility time, with a focus on local or global maximal coverage, depending on the application goals. We have already provided an example model for ground coverage calculation in Equation (1).

The choice of a suitable performance metric is very important and, naturally, application-dependent. For the space segment, some of the commonly known metrics are the Dilution of Precision (DOP)-based metrics, such as GDOP [139] and Time Dilution of Precision (TDOP) [140], and the system level metrics, such as $C/N_{0}$, SNR and Signal-to-Interference-plus-Noise Ratio (SINR).

It should be noted that such metrics are not exclusive to the space segment. In order to avoid repetition, we divide the metrics between the three LEO segments (see Figure 1) when a particular metric is used in multiple segments. For example, as DOP metrics, such as GDOP and TDOP, are widely used in user-segment optimization, we will describe those metrics in Section 8, while the other metrics such as SNR, Signal-to-Interference-plus-Noise Ratio (SINR), and $C/N_{0}$, used widely for all three segments, are presented here.

The most basic definition of SNR is the ratio of signal power to noise power, often expressed in decibels, as seen in Equation (10). Here, $P_{\cdot }$ denotes the power of the signal or noise in watts, measured at equivalent times and within the same system bandwidth.

(10) $${\mathrm{SNR}}_{dB}=10\log_{10}\left(\frac{P_{signal}}{P_{noise}}\right)$$

SINR is a very similar metric to SNR. It is often expressed in decibels (dB), as in Equation (11). Similarly to Equation (10), $P_{\cdot }$ denotes power in watts.

(11) $${\mathrm{SINR}}_{dB}=10\log_{10}\left(\frac{P_{signal}}{P_{interference}+P_{noise}}\right)$$

$C/N_{0}$, while close to SNR, is a much more commonly used metric for applications regarding LEO constellations. It is usually expressed in decibel-Hertz and refers to the ratio of the carrier power $P_{carrier}$ to the noise power $P_{noise}$ per unit bandwidth. It is calculated as in Equation (12), where notation from Equations (10) and (11) carries over in terms of units and definitions.

(12) $$C/N_{0}dB-Hz=10\log_{10}\left(\frac{P_{carrier}}{P_{noise}}\right)$$

Minimizing the costs is a logical objective for any commercial service; for a straightforward example, using a minimal number of satellites operating at low altitudes can increase the real-life feasibility of applications, as those parameters directly reduce the manufacturing and launch costs. However, the trade-off between constellation altitude and coverage depends on application. With a lower altitude, the satellite signal reaches the receiver at a higher SNR, but more satellites are needed to provide the coverage. As for a mathematical explanation, we have already presented a cost model from [123] in Equation (7).

Another significantly more complex metric that is required for the optimization process of most advanced methods, such as the Machine Learning (ML)-based approaches, is the application-specific objective function. This metric has different names and serves different purposes depending on the literature area; some examples are the ’fitness function’ (for evolutionary algorithms), ’the reward function’ (for RL-based methods), and ’the cost function’ (for the dynamic optimization methods). Such functions are typically designed to include a high number of non-linearities that best represent the important system characteristics based on the application. With regards to the space segment, an objective function can simply be any of the already mentioned metrics (i.e., $C/N_{0}$ for GNSS or LEO-PNT applications as well as for high communication throughputs), some combination of them (i.e., $C/N_{0}$ and GDOP), or completely custom crafted as in [20,74]. As an example, we present Equation (13), which defines an objective function f(x) that combines the $C/N_{0}$ and the launch cost $C_{launch}$ using some weights $w_{1}$ and $w_{2}$, where *x* is the vector of the required parameters for the calculations. Note that in a typical ML-based method, these weights are fine-tuned by the optimization process, but they can also be tuned manually, which is typical for evolutionary algorithms.

(13) $$f\left(x\right)=w_{1}C/N_{0}+w_{2}C_{launch}$$

### 6.8. Applicable Optimization Methods for Space Segment

Looking back at Table 2, we can identify the commonly used constellation optimization methods as:

- Brute-force approaches, such as the MC method (typically used to evaluate constellation designs together with theoretical modeling approaches [141,142]);
- Evolutionary algorithms, such as GA [21,74,143,144] and PSO [20,55];
- SA methods [21,143,145];
- ML-based methods [22,146];
- Other methods: in addition to the above-mentioned methods, there are also some advanced algorithms that have evolved from the previous ones, such as MOGA [19] or HRPSO [55], that combine the optimization process with analytical methods to improve or expand the optimization performance.

In the remainder of this subsection, we will provide overviews for the general versions of the most common methods.

#### 6.8.1. Genetic Algorithm (GA)

GA, originally introduced in [147], is an evolutionary algorithm that is used to find solutions to optimization problems, which relies on biologically inspired genetic operators: cross-over, mutation, and selection. We provide the steps of the plain GA in Algorithm 1. However, it should be noted that numerous variants of GA exist, such as an expansion to the multi-objective optimization problems (MOGA [52]) or the ones that follow specific operator criteria, for example, the selection criteria seen in NSGA-II [53], which we will cover in more detail at the ground segment.

Although we believe Algorithm 1 is sufficient as an overview, how to apply genetic operators in context can be confusing. To clarify what this means from a constellation optimization perspective, we provide the following example.

The population represents the collection of possible constellations, individuals represent particular constellations, and the genomes represent the parameters of a particular constellation. The cross-over operation determines the parameter vectors of the offspring from the parameters of the parents, as per the biological counterpart. Thus, if we consider the genome consisting only of the altitude and number of satellites, the offspring would have the number of satellites from the first parent and altitude from the second. Following the cross-over, the mutation operator introduces diversity to the population by mutating the genes of the offspring with some probability. Again, a very simple arbitrary example would be to have the mutation operator increase the altitude by 50 km with a 40% chance, reduce it by 20 km with a 40% chance, and have no changes with 20% chance.
**Algorithm 1 **GA**Step 1:** Population InitializationInitialize the population with *N* individuals, based on problem range and constraints.**Step 2:** Evaluate the Fitness FunctionEvaluate the value of the fitness (objective) function for each individual in the population.**Step 3:** Apply SelectionSelect individuals from the population using some selection criteria. A simple example is selecting randomly, but other selection metrics can be used depending on application.**Step 4:** Apply Cross-OverApply the cross-over genetic operator to the parents (i.e., produce a total of $\frac{N}{2}$ off-springs from the parents, with parameters carrying over from either parent with some probability).**Step 5:** Apply MutationApply the mutation genetic operator to the parents and off-springs (i.e., change genomes of an individual according to some joint probability of the mutation happening, what parameter to change, and how it changes)**Step 6:** TerminationRepeat steps 2–5 until a termination criteria is met (i.e., total iterations, change in optimality metric, etc.).

The last point of discussion regarding GA is the overhead of the method. While GA, like all the other evolutionary algorithms, requires a population that interacts with the environment (which naturally makes it an online method), it is typically applied to optimization problems via simulations in an offline manner. This is especially true in the case of constellation optimization, where parameters such as the number of satellites, the satellite inclination angles, etc., are almost impossible to modify once a satellite is launched on the orbit. As a result, the significance of the overhead comes from the required time and the computational complexity of the optimization method; both of which are primarily determined by the utilized software simulator and its computational complexity rather than by the optimization method itself. However, since an implementable optimization result obtained via GA will require a fairly complex simulation, the method’s overhead is difficult to generalize and must be examined case by case on a per-implementation and per-scenario basis.

#### 6.8.2. Particle Swarm Optimization (PSO)

PSO, originally introduced in [50], is another popular evolutionary algorithm that iteratively optimizes a problem by improving upon candidate solutions with respect to given metrics. The set of possible solutions are called the swarm and particular candidate solutions are called the particles, which have position and velocity vectors for each dimension of the particle (which can be defined as parameters of interest). Each particle’s movement is influenced by both its individual best and the best within the swarm, which is expected to move the swarm toward an optimal solution.

Algorithm 2 summarizes the PSO algorithm, and we provide the velocity update equation in Equation (14) and the position update equation in Equation (15), both of which are given for particle *i* and dimension *d*. *t* refers to time (or iteration), $w_{1}$, $w_{2}$, and $w_{3}$ are scalar weights, *v* refers to the vehicle speed vector in 3D space, x refers to the current position (vector form), $p_{i}$ is the best known position of particle *i*, $p_{g}$ is the best known position in the swarm, and ||·|| is the Euclidean metric or the distance between vectors.

(14) $$v_{i,d}\left(t+1\right)=w_{1}\left|\right|v_{i,d}\left(t\right)||+w_{2}\left|\right|p_{i}-x_{i,d}\left(t\right)||+w_{3}\left|\right|p_{g}-x_{i,d}\left(t\right)\left|\right|$$

(15) $$x_{i,d}\left(t+1\right)=x_{i,d}\left(t\right)+v_{i,d}\left(t+1\right)$$

Similarly with the GA, the PSO also have variants; the straightforward one is the expansion to the multi-objective optimization MOPSO [54]. More complex improvements work by including extra heuristics or mathematical strategies to guide the particle’s through the search space in a more informed manner; an example seen in [55] uses different re-sampling methods in a hybrid manner to achieve this, similar to particle filtering applications.
**Algorithm 2 **PSO**Step 1:** Swarm InitializationInitialize the swarm with *N* particles, based on problem range and constraints. Randomly initialize their positions and velocities within the search space.**Step 2:** Fitness Function EvaluationEvaluate the value of the fitness (objective) function for each particle in the swarm. Update the particle best and swarm best for each particle.**Step 3:** Position and Velocity IterationIterate the position and velocity of each particle according to the Equations (14) and (15). Enforce limits if any particle goes out-of-bounds of the search space.**Step 4:** TerminationRepeat steps 2–3 until a termination criteria is met (i.e., total iterations, change in optimality metric, etc.).

As another evolutionary algorithm, PSO has almost identical overhead properties as GA. However, while the traditional PSO is typically faster to converge to a solution compared with GA [148], it also has certain ’pitfalls’ that can prevent or significantly alter the required number of iterations to reach a solution (i.e., particles getting stuck in the local minimum or maximum).

#### 6.8.3. Simulated Annealing (SA)

SA, while similar to evolutionary algorithms, is a meta-heuristic algorithm that specializes in finding an approximation to the global optimal solution in large search spaces. It was originally introduced in [48] and was inspired by the annealing procedure of metalworking. Similar to evolutionary algorithm counterparts, variants that expend the method exist, such as MOSA [51].

SA is ideal for computationally hard optimization problems where exact algorithms prove infeasible; even though it only achieves an approximate solution, it is typically enough for many practical problems. In general, SA algorithms assume a system initialized with a positive temperature and a starting solution (represented by point *X*, typically a vector of parameters of interest). At every iteration, the algorithm randomly (or heuristically, in improved versions of the algorithm similar to PSO variants) selects a point close to the current one and decides to accept it according to the acceptance criteria, which is typically a probabilistic objective function that depends on the system temperature. The system’s temperature gradually decreases, and the final solution is considered an approximation to the global optimal. We present the procedure in Algorithm 3.
**Algorithm 3 **SA**Step 1:** System InitializationInitialize the system with an initial temperature $T_{0}>0$ and generate a starting point $X_{0}$, which will also be initialized as the best known point $X^{*}$.**Step 2:** Move to NeighborsCheck a neighboring point $X_{s}$ and decide if the neighbor is ‘better’ according to an acceptance criteria. The acceptance criteria must have some probability of accepting ‘worse’ neighbors related to the temperature of the system (higher-temperature systems have a higher probability of accepting ‘worse’ neighbors). If the neighbor is accepted, move to it and assign $X_{s}=X^{*}$; otherwise, stay in the current point.**Step 3:** Enforce Annealing ScheduleRepeat Step 2 until an ‘equilibrium condition’ is satisfied (in practicality, this condition can be quite complex or very simple. For example; after a number of iterations, or after every point change). When this condition is met, decrease the temperature of the system and move back to Step 2.**Step 4:** TerminationRepeat steps 2–3 until a termination criteria is met (i.e., total iterations, minimum temperature, etc.).

Overhead-wise, SA is similar to both GA and PSO in the sense that it is typically applied in an offline manner while inherently being an online method. However, since the main strength of the SA method is finding an approximate solution to the global optimum when the exact methods fail, its time and computation properties are mainly an issue of feasibility rather than overhead. Typically, the overhead of the SA method is not a point of critical consideration when SA reaches an acceptable solution, which, in practice, is fine-tuned with the initial starting solution to shorten the computation time.

#### 6.8.4. Theoretical Modeling and ML-Based Approaches

Technically, theoretical modeling can also be seen as an optimization technique because it is included in steps that relate to the optimization process as a whole, such as a necessity in methods and metrics as criteria, as we have briefly touched upon in this section. Alternatively, they can be used as a system model, typically within simulations (i.e., a brute-force approach, MC Optimization, solvers, etc.). Needless to say, it would not be possible for us to cover an extensive amount of models in detail, and covering examples makes little sense as they serve as optimization methods only for the particular case they model. Thus, we believe the models we include in our discussions with respect to the methods and metrics are sufficient to demonstrate the role of modeling in an optimization context related to the segments.

Similarly, ML-based methods are tricky and difficult to explain briefly, as the networks themselves are designed in a case basis. The study we have cited in Table 2 with regards to ML is [22], where the authors utilize a DNN to modulate and demodulate the signal at terminal and relay nodes to optimize constellations in two-way relaying with physical-layer coding. Regarding their work, the details are complex and are not suited for overview in this paper. However, we will provide an overview about the general NN structure in its stead.

An NN consists of layers, which are mathematical manipulations characterized by the neurons (or nodes) within the layer. A neuron is just a place where computation happens, inspired by the neurons in the human brain that fire when stimulated. In a neuron, input data matrix *X* is multiplied by the weight matrix *W* and is propagated through an activation function Θ(·) to generate the output matrix *Y*:

(16) $$Y=\Theta (W^{T}X)$$

This operation assigns significance to inputs with regard to the task the network is trying to learn and determines whether and to what extent that input should progress further through the network to affect the ultimate outcome. This process is called ’training’. While, in practice, it is limited by the available data, in theory, it does not terminate until the operation stops altering the weights used in the operation according to an optimality criterion (typically, a gradient descent is utilized for this purpose).

**Figure 6:**
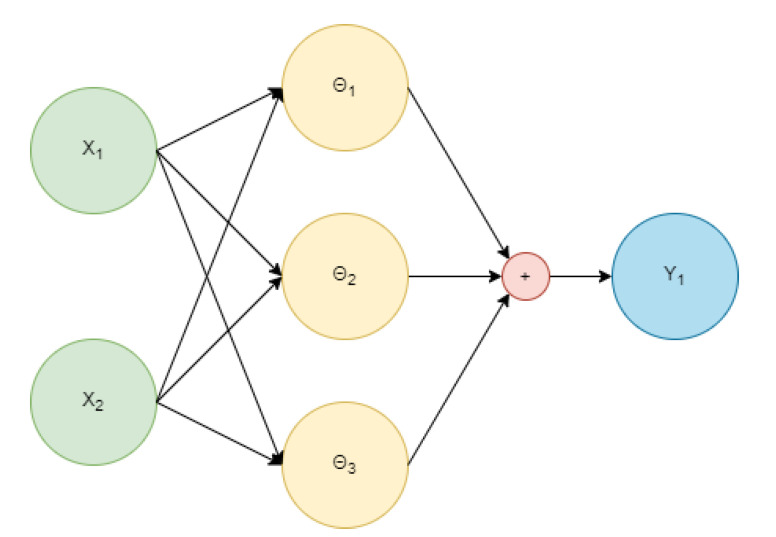
An example of a simple NN, with 1 hidden layer consisting of 3 neurons.

An NN layer is a row of such neurons that turn on or off according to their activation functions, as the input is fed through the network. Each layer’s output is simultaneously the subsequent layer’s input, starting from an initial input layer receiving the data. An extremely simple NN with only one layer is shown in Figure 6 to serve as an example.

A DNN, as the name suggests, is a type of NN. The adjective ’deep’ refers to the use of multiple layers in the network. Typically, a DNN utilizes a very large number of layers but is a forward network. This means that the NN architecture does not loop or back-propagate. Other types of NNs utilize such architectures, such as a CNN or Recurrent Neural Network (RNN). We will cover the RNN variant in the ground segment; however, we will refrain from presenting an overview of the other ML-based optimization methods.

In the context of data-driven approaches (which includes NN and its variants), online and offline optimization refer to their counterparts in learning theories, namely the online learning and offline learning. Online learning indicates that the learning takes place as the data come in, while the offline learning indicates the existence of a static dataset from which the learning is performed. ML-based methods can be constructed to operate with either of the two learning methods, or, in many cases, simultaneously, with both methods. The learning method imposes additional constraints to the feasibility of the method (the obvious example being the computation time constraint for online learning case, which operates in real-time). As with the other complex cases, these additional constraints need to be taken into account when an implementation decision is made to use ML-based methods in practice.

### 6.9. Method Comparisons for the Space Segment

A comparison of the optimization methods presented in Table 2 and explained throughout this section is presented in Table 9 in terms of their applicability for LEO space-segment-related optimizations. We compare the methods under three categories, which we loosely define as:

- **Convergence Speed:** Overall performance in terms of the time it takes for the method to reach a solution and computational complexity. For data-driven methods, this roughly relates to how big of a dataset is required.
- **Global Convergence:** Overall performance in terms of finding the global optima. Many methods can get stuck in a local optima, especially for complex optimization problems.
- **Large-Scale Applicability:** Overall performance in terms of how many different problems can be solved utilizing the method and how simple it is to formulate optimization problems (or find/create datasets) to use the method, with respect to the denoted segment.

An important remark is about the theoretical modeling: we intend for the method of theoretical modeling to represent our interpretations of the approach in general rather than the individual performance of models. As a result of this ’simplification’, the corresponding row in the table reflects the above-defined categories as a generalization.

## 7. Ground-Segment Optimization Aspects

The main optimization problem with respect to the ground segment refers to the optimization of the number and placement of ground stations all over the Earth in such a way that they are able to offer good monitoring, management, and control of all LEO satellites in the sky. However, currently, there are very few studies in open access describing optimization methods for LEO ground segments. In contrast, many such studies exist for MEO GNSS systems, and it is to be expected that some of the optimization mechanisms previously proposed for GNSS are also applicable in the LEO context. As a result, in this section, we will present the metrics and methods that have seen successful in either case.

### 7.1. Optimization Metrics Related to the Ground Segment

The study in [149] looked at the coverage as the optimization metric in the context of Beidou GNSS and aimed at having the same satellite in view for a predetermined interval of time by at least four ground stations (thus, they called this metric a ‘quadruple coverage’ metric). Although the study compared only two deployments based on 18 ground stations each (thus, there was no complex optimization problem involved), it served as a good example of utilizing sky coverage percentage as a metric for the general optimization goal of maximizing coverage in GS planning.

The sky coverage is a metric such that it requires a mathematical model to operate with, which is typically constructed slightly differently depending on the objective of the analysis. In [149], the authors defined it as *‘the percentage of observing time to a certain satellite by at least M stations within a specified time period’* and formulated it from the GS visible curve to the satellite using simplified geometric calculations.

The problem of finding the best location of a GS was addressed in [150] in the context of MEO Satellite-Based Augmentation System (SBAS). The target metrics for the geographical location optimization were the scintillation fade depth, the GDOP, the number of visible satellites, the ionospheric delay, and the rainfall attenuation, which were all varying metrics indicating the signal-availability level. While the problem was formulated as a multi-modal optimization problem, no final optimal solution was given, but rather a trade-off criterion by criterion was presented.

The number of satellites and GDOP are related metrics; as the number of satellites in view increases, the value of GDOP decreases, which ensures good accuracy. In [150], the authors followed the GDOP calculations from [151] and used GDOP as the optimization metric. The GDOP is defined in Equation (18) based on the least square solution seen in Equation (17).

(17) $$\Delta y={\left(H^{T}H\right)}^{-1}H^{T}\Delta x$$

(18) $$\mathrm{GDOP}=\sqrt{tr{\left(H^{T}H\right)}^{-1}}$$

Above, Δy is the position offset as obtained from the least squares solution of the linearized pseudorange measurement model of the system, *H* is the satellite geometry matrix, and Δx is the net error in the pseudorange value. tr(.) refers to the trace operation.

Note that, in order to formulate the set of equations whose least squares solution provides the GDOP, a modeling process is required that expresses the geometry between the users (for our case, this is the geometry between the GS and the satellite). In addition, modified versions of GDOP such as the weighted GDOP [151] also exist, which include a weight matrix *W* to the model, as in Equation (19).

(19) $$WGDOP=\sqrt{tr{\left(H^{T}WH\right)}^{-1}}$$

Following the optimization metric definitions from [150], the scintillation fade depth $FD_{s}\left(p\right)$ at level ρ is calculated in dB, as summarized in Equation (20), where $\sigma_{s}$ is the standard deviation of the signal for a desired period, t(ρ) is the time percentage factor, and ρ is the percentage availability within the range 0.01%<ρ<50%.

(20) $$FD_{s}\left(\rho \right)=t\left(\rho \right)\sigma_{s}$$

(21) $$t\left(\rho \right)=-0.061{\left(\log_{10}\rho \right)}^{3}+0.072{\left(\log_{10}v\right)}^{2}-1.71\log_{10}\rho$$

For other optimization metrics from [150], the authors utilized the model by Klobuchar (a detailed explanation can be found in [152]) for the ionospheric delay and the International Telecommunication Union—Radio-communication unit (ITU-R) model for the rainfall attenuation.

Another ground-segment optimization problem was addressed in [26] in the context of generic high-throughput satellites (with unspecified orbit altitudes) and using a machine-learning optimization via deep learning with Long Short-Term Memory (LSTM). The principle in [26] was to allow redundant ground stations (or gateways) in such a way that the effect of rain attenuation was mitigated during the ground-to-satellite and satellite-to-ground communications. The rain attenuation was modeled by ITU-R models in this study as well, and the optimization metric was the Carrier to Interference plus Noise Ratio (CINR), which is expressed in Equation (22) at a discrete sample (or time instant) *n* and for the *i*th gateway, where $|h_{i}\left[n\right]|$ is the wireless channel amplitude at sample *n* for the *i*th gateway, $\sigma_{s}$ is the noise variance for the ground-to-air channel, $I_{i}$ is the interference level affecting each gateway site, and $P_{i}$ is the average transmission power.

(22) $${\mathrm{CINR}}_{i}\left[n\right]=\frac{P_{i}{\left|h_{i}\left[n\right]\right|}^{2}}{\sigma_{i}+I_{i}}$$

Yet another ground-segment optimization in the context of optical ground-to-satellite links was investigated in [153], targeting the Link Outage Probability (LOP) as the optimization metric and considering three brute-force methods and a fixed maximum number of ground stations (namely 66) with fixed locations. The optimization output was a subset of ground station locations that offer the lowest LOP, which is defined as a function of *M*-out-of-*N*-availability given by Equation (23), where *M* refers to the smallest number of available GS required at a given time, *N* refers to the total number of GS in the network, $A_{i}$ is the availability of *i*th GS, and $q_{i}=1-A_{i}$.

(23) LOP=∑i=0M−1NiAi×qN−i

### 7.2. Applicable Optimization Methods for Ground Segment

Looking back at the state-of-art methods from Table 2 and the example studies from the previous subsection, which mostly utilize traditional methods for simple analysis, we can identify the main applicable optimization methods for the ground-segment optimization as NSGA-II, LSTM, and Iterative Ground Station Deployment based on Marginal Revenue Maximization (IGSD-MRM). In this subsection, we will provide an overview for these methods.

#### 7.2.1. Non-Dominated Sorting Genetic Algorithm II (NSGA-II)

NSGA-II, originally introduced in [53], is an improvement over MOGA [52], which has three characteristics; i) it uses an elitist principle; ii) it uses an explicit diversity-preserving mechanism (called crowding distance); iii) it emphasizes non-dominating solutions. We provide the steps of NSGA-II in Algorithm 4. It is to be noted that the individuals that form the population are the possible solutions that we are interested in. For example, for the GS planning problem, a possible implementation is to define a GS network distributed randomly in the area of interest as an *individual*; such *individual* will be made up of a set of four vector parameters, namely the 3D position, the elevation angle, the number of GS, and the overall manufacturing cost. The parameters of an *individual* are thus not limited to scalars only, but they can be vectors or a set of vectors themselves. Furthermore, as NSGA-II is an advanced variation of GA, it has identical overhead properties with it.

**Algorithm 4 **NSGA-II
**Step 1:**Population InitializationInitialize the population with*N* individuals based on problem range and constraints.**Step 2:** Value EvaluationEvaluate the value of the objective function for each individual in the population.**Step 3:** Non-Dominated Sort with Crowding DistanceSelect $\frac{N}{2}$ individuals to be the ’parents’ in the next generation, according to the following criteria:   **a.** Prioritize individuals with better non-domination criteria.   **b.** Between individuals of similar non-domination criteria, prioritize individuals with lower crowding distance.**Step 4:** Cross-OverApply the cross-over genetic operator to the parents (i.e., produce a total of $\frac{N}{2}$ off-springs from the parents, with parameters carrying over from either parent with some probability).**Step 5:** MutationApply the mutation genetic operator to the parents and off-springs (i.e., change parameters of an individual according to some joint probability of the mutation happening, what parameter to change, and how it changes)**Step 6:** TerminationRepeat steps 2–5 until a termination criterion is met (i.e., total iterations, change in optimality metric, etc.).


#### 7.2.2. Long Short-Term Memory (LSTM)

**Figure 7:**
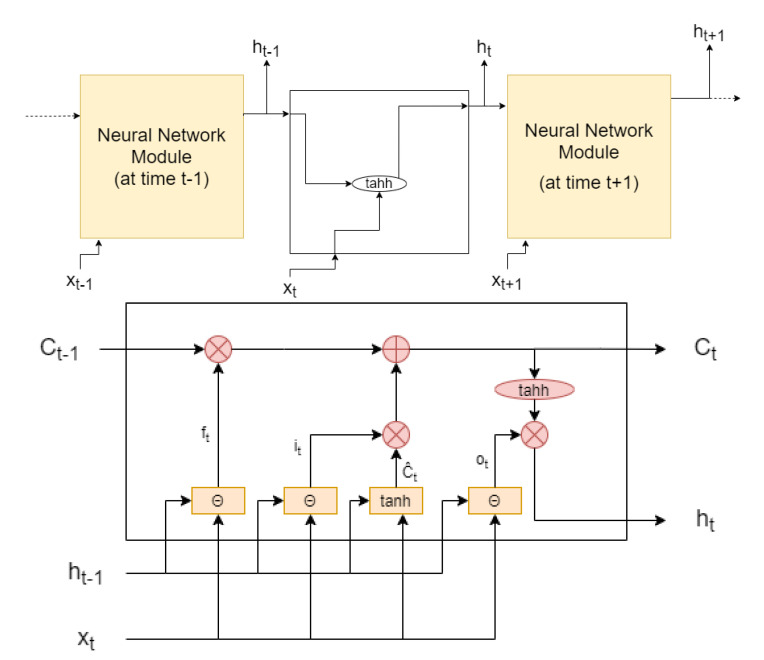
Upper Plot: The Block Diagram of a Repeating Module in a Standard RNN, which is the generalization of LSTM. Note that the modules are identical in structure, and the time propagation comes from the inherent self loop of an RNN. Lower Plot: Schematic Diagram of a Standard LSTM Module.

LSTM, originally introduced in [154], is a special kind of RNN that is particularly good at learning long-term dependencies within a system. All RNN have the form of a chain of repeating modules of NN. In a standard RNN, this repeating module has a very simple structure, such as a single tanh layer. The upper plot in Figure 7 illustrates this structure, where $x_{t}$ is the input to the network at time *t* and $h_{t}$ is the output of the network at time *t*.

LSTM also has this chain-like structure as it is a RNN itself, but the repeating module has a different, more complex structure seen in the lower plot of Figure 7. In the lower plot of Figure 7, *x* and *h* are also the inputs and outputs of the network, respectively, $C_{t}$ is the cell state of the network at time *t*, Θ is a chosen activation function (typically the sigmoid function), the circular red elements represent component wise operations, and the rectangular yellow elements represent the network layers who perform operations according to Equations (24)–(29). In these equations, *f* represents the output of the ’forget’ layer, *i* represents the output of the ’input’ layer, *o* presents the output of the ’output’ layer, and $\hat{C}$ represents the output of the cell state estimation layer. The variables $W_{\cdot }$ and $b_{\cdot }$ refer to the weight matrix and bias of the layer, noted by their subscripts (o,i,f,c), respectively. The subscript *t* refers to time, and the other subscripts *f*, *i*, *c*, and *o* denote the different layers, as explained above.

(24) $$f_{t}=\Theta \left(W_{f}\left[h_{t-1},x_{t}\right]+b_{f}\right)$$

(25) $$i_{t}=\Theta \left(W_{i}\left[h_{t-1},x_{t}\right]+b_{i}\right)$$

(26) $${\hat{C}}_{t}=tanh\left(W_{c}\left[h_{t-1},x_{t}\right]+b_{c}\right)$$

(27) $$o_{t}=\Theta \left(W_{o}\left[h_{t-1},x_{t}\right]+b_{o}\right)$$

(28) $$C_{t}=f_{t}c_{t-1}+i_{t}{\hat{C}}_{t}$$

(29) $$h_{t}=o_{t}tanh\left(C_{t}\right)$$

We remark that the Equations (26) and (29) use the tanh(·) operator only as an example of an output activation function to emphasize that such operator is typically a different one than the other layer’s activation functions. Any other activation function can also be used in its place for the output depending on the application, but sigmoid and tanh are typical for LSTM.

As for the overhead, studies such as [155,156] provide optimal LSTM designs that boost the performance and reduce the overhead for their cases of interest, but for the general purpose, it remains a necessity to perform per case evaluations as any other ML-based method.

#### 7.2.3. Iterative Ground Station Deployment based on Marginal Revenue Maximization (IGSD-MRM)

Unlike NSGA-II and LSTM, which are relatively general and proven methods in the literature, IGSD-MRM is a recent method proposed in [27]. It is an iterative optimization method for the GS planning problem in the context of LEO satellite networks. The optimization goal is to maximize the system throughput capacity $C_{tot}$, which is defined by Equation (30), where $C^{UL}$ is the user link capacity, $C^{FL}$ is the feed link capacity, and *D* is the capacity of the LEO network. The subsets $s_{m}$ and $b_{n}$ represent $m^{th}$ satellite and $n^{th}$ beam, respectively. Both $C^{UL}$ and $C^{FL}$ are calculated from Equation (31), where $B_{c}$ is the link bandwidth and M(.) is the mapping from SNR to spectrum efficiency under the DVB-S2X transmission scheme.

(30) $$C_{tot}=\sum_{m=1}^{M}\sum_{n=1}^{N}min\left(C_{s_{m},b_{n}}^{UL},C_{s_{m},b_{n}}^{FL},D_{s_{m},b_{n}}\right)$$

(31) $$C=B_{c}M\left(SNR\right)$$

The following steps summarize the IGSD-MRM optimization procedure, adapted from [27]:

Step 1: Divide the area of interest into smaller grids. In [27], the authors divide the entire world into $1^{\circ }$ latitude by $1^{\circ }$ longitude and granularity grids.
Step 2: Calculate the traffic demands of the satellite. In [27], the hotspot model is assumed for the traffic model, and the notation is such that *F* denotes the traffic demand for the grid and $F_{s}\left(X_{i}\right)$ denotes the traffic demand for satellite *s* at position $X_{i}$.
Step 3: Calculate the marginal revenue for each possible GS deployment using the following:
  - Calculate the spherical distance between the satellite position and the GS, then calculate the corresponding geocentric angle.
  - Calculate the path loss, link budget, and spectrum efficiency.
  - Calculate the achievable link capacity.
  - Take the gridded revenue as $min(C\left(X_{i}\right),F_{s}\left(X_{i}\right))$
Step 4: Choose the location with the maximal marginal revenue as the GS deployment location.

As for the overhead, the original paper [27] does not include a detailed analysis about computation complexity/convergence time for the method, so the literature is in need of more implementations that utilize IGSD-MRM before we can provide a generalized insight on this aspect.

### 7.3. Method Comparisons for the Ground Segment

Finally, we compare the discussed optimization methods throughout this section in Table 10. Note that we intend for the theoretical modeling entry to be a generalization for modeling and brute-force approaches rather than as an indicator of the performance of particular models.

## 8. User-Segment Optimization Aspects

The user segment can be seen as the largest segment in terms of optimization challenges, as it has the highest number of metrics and parameters related to the system optimization, and they are highly scenario- or application-specific. Even when narrowed down to the area of transportation and UAV, any optimization problem that involves a LEO satellite system is included in this user segment, and therefore, there is still a wide pool of optimization problems to be formulated and addressed. In this section, we present a few selected problems in order to focus on the user perspective, as previously presented in Table 2, but it is also worth noting that we can also optimize some aspects of the LEO space- and ground-segments from the user-segment point of view.

A concrete example is the satellite-selection problem when it is expected to have a huge number of satellites in the sky. This satellite-selection problem refers to the problem of selecting the best subset of available (or visible) satellites at the receiver in order to attain specific communication, positioning, or sensing targets. This problem relies on the hypothesis that having a bigger constellation does not directly mean one can achieve a better performance metric, for example, in terms of DOP [5]. If the constellation is excessively large, the information provided by two or more satellites that are close to each other is highly correlated and thus not adding value when increasing the number of processed satellite signals, and we can consider that the satellite system would be overpopulated in such cases. In addition, the receiver processing complexity increases with the number of satellite signals to be processed; therefore, selecting the best satellites to process is important. In this case, the optimization problem becomes figuring out which of those satellites we have in view are most representative and, for example, which configuration would provide the best geometric distribution among other factors. We can optimize and select those satellites with the best score metrics (individually analyzed or combined) using the methods and metrics from Table 2.

**Figure 8:**
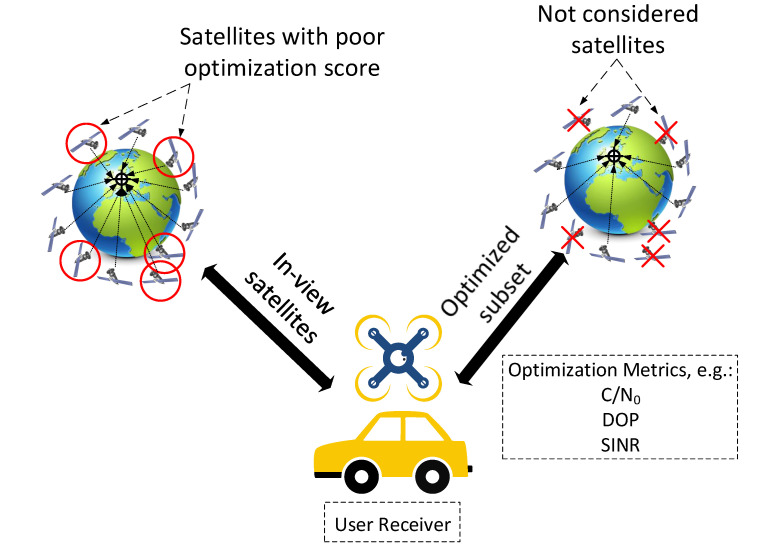
Example of an Optimization Problem from the User-Segment Perspective: The satellite-selection problem, i.e., the total number of satellites in view is to be reduced to an optimal subset according to application-specific optimization metrics.

Figure 8 shows an illustration of this optimization from the user perspective in a GNSS scenario. In Figure 8, the satellites in view depicted with a red circle are redundant (e.g., DOP contribution is bad, elevation is very low, etc.). Thus, the best approach would be that these satellites are not to be further considered, but only a subset of the available satellites (e.g.,the one with some high scores according to the defined performance metrics) is to be used for further processing.

In the rest of this section, we present various metrics and parameters that are used and/or adjusted in order to keep a good general performance of the system, and we present an overview of various optimization methods that have seen used in applications in recent years. This section addresses the optimization problems from the user-segment perspective.

### 8.1. Optimization Metrics Related to the User Segment

Of the metrics seen from Table 2 related to the user segment, some of them have already been presented in previous sections; for example, we presented GDOP in Equation (18), which is also utilized in the user segment for the satellite-selection problem. Thus, we will present an overview of the metrics that have not yet been covered in the other segments here. We will also omit basic parameter metrics as the definition of the parameter is sufficient enough of an explanation for these metrics, such as the antenna elevation in satellite selection, travel time and distance in UAV data acquisition or latency, and the number of ping pongs in the handover planning.

It is best if we start with introducing metrics similar to GDOP that are not dependent on the models utilized in the system. One such metric is the TDOP, which is closely related to GDOP as another DOP metric. It is presented in Equation (32), where the notation from Equation (18) carries, and 4 refers to the fourth element of the trace vector, which represents time, thus the name. Other DOP metrics also exist in the literature, all of which are covered in earlier studies in detail, such as in [157].

(32) $$\mathrm{TDOP}=\sqrt{tr{\left(H^{T}H,4\right)}^{-1}}$$

Another metric that is seen commonly in network performance evaluation is the Bit Error Rate (BER), which is a unitless metric that is defined as in Equation (33).

(33) $$\mathrm{BER}=\frac{\mathrm{number}\;\mathrm{of}\;\mathrm{bit}\;\mathrm{errors}}{\mathrm{total}\;\mathrm{number}\;\mathrm{of}\;\mathrm{transferred}\;\mathrm{bits}}$$

BER is a very simple error metric that simply looks at the error percentage in bits. Other similar metrics from Table 2 that look at the error percentage of a particular parameter are the handover and failure percentages, which can be written in terms of Equation (33) by replacing bits with the parameter of interest. However, one should also note that these types of metrics can also be utilized together with models, as seen with BER in [36].

A slightly different but very informative performance metric we still need to address is the Root Mean Square Error (RMSE), which is a widely utilized error metric in data-driven optimization approaches. RMSE is a comparative metric that provides a performance relative to something else; ML methods typically use a test dataset for this purpose, and in the case of using RMSE as a tracking error, it is simply the actual trajectory of the tracked target. We provide the RMSE calculation in Equation (34), and we also provide a similar comparative error metric called Mean Absolute Error (MAE) in Equation (35). In both equations, *n* refers to the total number of data points, $y_{i}$ refers to the prediction in point i=1,…,n (i.e., the output of a NN or the estimated point in trajectory), and $x_{i}$ refers to the true value in point *i* (e.g., the labels of the data in a NN or the point in the real trajectory for tracking). More examples of comparative error metrics can be found in the literature, for example, in [158].

(34) $$\mathrm{RMSE}=\sqrt{\frac{1}{n}\sum_{i}{\left(y_{i}-x_{i}\right)}^{2}}$$

(35) $$\mathrm{MAE}=\frac{1}{n}\sum_{i}\left|y_{i}-x_{i}\right|$$

As for the other metrics that include models, the user segment also utilizes coverage as a metric, which we have discussed in the space and ground segments. Another such metric is the network energy calculation for the UAV data acquisition problem. We will provide an example by following the network energy consumption model from [36], where a wireless sensor network is optimized and energy consumption is one of the three major metrics utilized. In [36], the authors model the total energy consumption of the network as in Equation (36), where $E_{ij}$ refers to the energy consumed for communication between node *j* and the channel head node *i*, $E_{iu}$ refers to the energy consumed for communication between the UAV node *u* and the channel head node *i*, and *N* refers to the total number of channel head nodes.

(36) $$E_{tot}=\sum_{i}^{N}\sum_{j}^{N_{i}}E_{ji}+\sum_{i}^{N}E_{iu}$$

$E_{ji}$ is modeled as in Equation (37), where $P_{t_{ji}}$ is the transmit power and $R_{ji}$ is the bit rate of the communication channel between node *j* and channel head node *i*. $E_{iu}$ is modeled similarly as in Equation (38) but between channel head node *i* and the UAV node *u*.

(37) $$E_{ji}=\frac{P_{t_{ji}}}{R_{ji}}$$

(38) $$E_{iu}=\frac{P_{t_{iu}}}{R_{iu}}$$

Lastly, a more arbitrary metric could be the overall complexity of a design. Given a satellite selection problem from the point of view of the user receiver, a first approach could be trying to reduce the complexity by reducing the number of satellites being tracked. In this sense, we can make use of DOP metrics such as GDOP and TDOP, received power strength such as $C/N_{0}$, or even simple parameter metrics, such as the satellite elevation from the user location, to optimize the number of satellites to be tracked.

In the literature, we can find some studies that utilize a similar idea but are typically applied to GNSS scenarios. For example, in [159], the authors proposed a DOP optimization based on TDOP, in which the subset of satellites with the best TDOP were used for obtaining the PNT solution. In [160], the authors proposed a method called the ‘Quasi-Optimal Technique’, in which more than four satellite DOP measurements are considered to optimize the satellite subset selection. The authors in [161] proposed a less computational intensive method compared to [160] but using a similar approach. In [162], the authors propose a weighted least-squares-based DOP algorithm. The algorithm uses different metrics, besides the DOP, in order to weight the final optimal subset of satellites. In [163], the authors propose a brute-force method, in which they compute DOP metrics for the different subsets of all satellites in view and take as the optimal subset those satellites with the lowest DOP metric. Finally, in [164], the authors analyze the optimal number of satellites to be selected until the DOP metric becomes saturated. Thus, including a higher number of satellites does not improve the metrics significantly.

### 8.2. Illustrative Simulation-Based Examples

**Figure 9:**
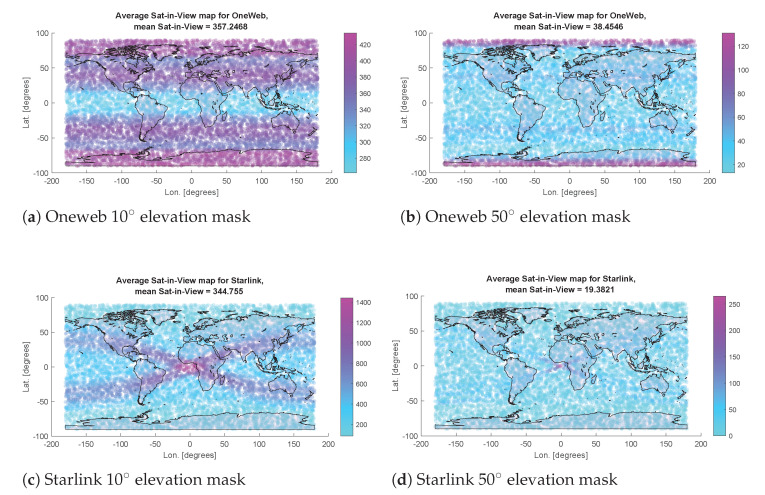
Example of Coverage Maps for Two Selected LEO Constellations: Oneweb (**top**) and Starlink (**bottom**), as number of satellites in view per Earth point for two different elevation masks: 10^o^ in the left-side plots and 50^o^ in the right-side plots.

**Figure 10:**
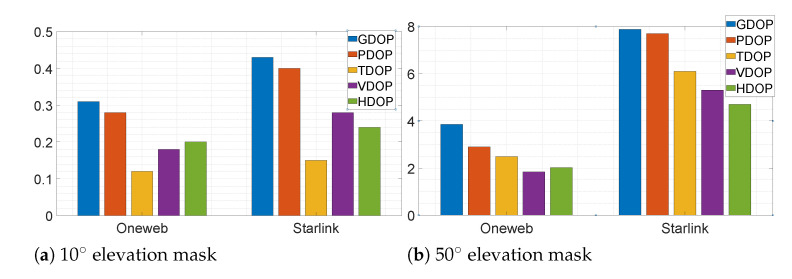
DOP metrics comparison for Oneweb and Starlink LEO constellations with two different elevation masks: (**a**) 10${}^{\circ }$ elevation mask and (**b**) 50${}^{\circ }$ elevation mask.

**Figure 11:**
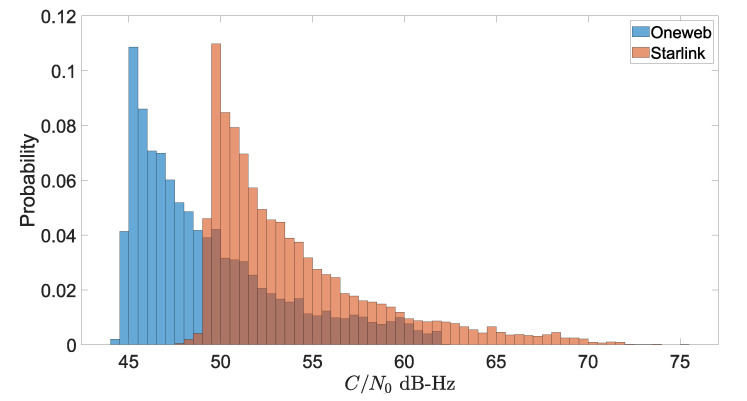
Example of $C/N_{0}$ distributions across the Earth points for LEO constellations Oneweb (blue) and Starlink (orange).

In order to show examples of some of the metrics described in the previous subsection, we have carried out some simulations with specific LEO constellations. Oneweb and Starlink LEO constellations were selected as more representative and promising among the future LEO mega-constellations. Figure 9 shows the coverage map for these two LEO constellations considering two different elevation masks, $10^{\circ }$ and $50^{\circ }$, respectively. Moreover, the average number of satellites in view in the whole Earth is also shown. We notice from Figure 9a,c that, in both OneWeb and Starlink constellations, when a small elevation angle is considered (e.g., $10^{\circ }$), the average number of satellites in view is very large, with more than 100 and more than 300 satellites in view for Oneweb and Starlink, respectively. These values were obtained considering the designed planned constellations, which will be operative in the coming years. The planned constellations are designed for having 7808 and 34,404 for Oneweb and Starlink, respectively, distributed at different altitudes and orbital planes configurations, with a Walker star constellation topology. The visibility conditions were configured as those satellites with a higher elevation angle than the chosen elevation masks (i.e., $10^{\circ }$ or $50^{\circ }$) from the user-location perspective. In addition, $10^{4}$ Earth user locations were considered for all the simulations in Figure 9, Figure 10 and Figure 11. To better model the orbits, a Simplified Deep Space Perturbation model (SDP4) [165] was used along with the Matlab Satellite Communication Toolbox. The SDP4 model accounts for the effects of the oblateness of the Earth and atmospheric drag effects in the satellite orbit, among other effects.

Figure 10 depicts DOP metrics, namely GDOP, Position Dilution of Precision (PDOP), TDOP, Vertical Dilution of Precision (VDOP), and Horizontal Dilution of Precision (HDOP), for Oneweb and Starlink constellations. From Figure 10, we can observe that both constellations offer a similar DOP performance. GDOP is <1 for both Oneweb and Starlink, respectively, but only when considering a low elevation mask (e.g., $10^{\circ }$). If the minimum elevation to consider visibility is increased, the amount of satellites contributing to the measurements is largely decreased, as well as its spatial diversity, negatively influencing to these DOP values. For example, GDOP measurements considering a high elevation mask (e.g., $50^{\circ }$) become approximately 12 in both constellations. The rest of DOP metrics are kept below 1 in both cases with a low elevation mask and much larger considering a high elevation mask. DOP metrics are examples of optimization metrics that can be inserted in user-segment optimization.

Finally, Figure 11 shows the $C/N_{0}$ probability distribution function for Oneweb and Starlink LEO constellations, respectively.

For the simulations, we considered a rural scenario with a Line of Sight (LOS) condition. This means that direct vision between user receiver and satellites was achieved, as well as low density of scatters and multipath components. To simulate a realistic satellite-to-ground channel in the described scenario, we used the freely available QuaDRiGa [166,167] framework. This channel model is a Matlab-based software developed by Fraunhofer HHI that enables the modeling of radio wireless channels by generating realistic radio channel impulse responses. The specific parameters the software uses to simulate the satellite channel models are described in [168], which, in turn, are modified versions of Third-Generation Partnership Project (3GPP)/ITU-R channel models.

From Figure 11, we can observe that the received $C/N_{0}$ from OneWeb satellites is about 5 dB lower with respect to the Starlink constellation. Oneweb typical $C/N_{0}$ is about 45 dB-Hz, while Starlink is 50 dB-Hz. The $C/N_{0}$ levels are mainly influenced by: (i) the carrier frequency used by each constellation and (ii) the altitude of the orbit. Even though both constellations use a similar carrier frequency, in Ku-band (e.g., 12 GHz), their orbital altitude and orbital configuration is different. While Oneweb satellites are to be at altitudes of about 1200 km, Starlink satellites are to be distributed in orbits closer to the Earth, between 300 km and 600 km, thus offering a better link budget than those placed at higher altitudes. This is the main reason for the difference between $C/N_{0}$ levels; nevertheless, the $C/N_{0}$ distributions, as seen from the histograms in Figure 11, are very similar, and they resemble an exponential distribution. Such information can be incorporated into an optimization problem, for example, by imposing the constraint of a minimum desirable $C/N_{0}$ at any Earth point or in a target region.

As we have seen in Figure 9, Figure 10 and Figure 11, we have many different metrics that can be used from the user perspective to optimize those satellites contributing more and in a better way to the final PNT solution.

### 8.3. Applicable Optimization Methods for User Segment

Besides the overlapping methods that we have already explained in Section 6 and Section 7, Table 2 identifies the main applicable optimization methods for our selected problems in the user segment as ML, RL, Multi-Agent Reinforcement Learning (MARL), and KF, as well as the more traditional approaches, such as search methods based on modeling and routing algorithms (Dijkstra, A*, etc.). In this subsection, we will provide an overview of these methods with the exception of the ML-based approaches (including RL and MARL), which are too complex for us to provide a brief mathematical overview in this paper. Although we will include these methods in our comparison discussions, for details regarding those methods and how they can be used with regards to the user segment in LEO networks, we encourage readers to refer to the cited studies [30,32,41,42].

Before we start with the optimization methods, it is worth briefly talking about some traditional methods. Although not exactly methods of optimization, some traditional methods are still used to address some of the problems we included in Table 2, at the very least, as a reference for more complex methods. Such a method is the ’Elevation Method’, which is a simple and straightforward method of satellite selection in the user segment. The method sorts the satellites using their elevation angles and keeps the *n* satellites with the largest values. If there are more satellites above the mask than there are tracking channels, the lowest elevation satellites are excluded. While such a method of removing the lowest satellites can increase the DOP-based performance metrics, it lacks other important factors, such as satellite health or weighting factors. Simply looking at the elevation angle discards the possibility of using these additional metrics. Similarly, the ’Longest Period Method’ focuses on the visibility of the satellites and chooses the one with longest time spans, which contains almost all of the negatives associated with the elevation method but with different metrics. Other methods, such as the ’Downdate Method’ proposed in [31] or the method utilized in [29], combine some of the traditional ideas together, such as applying a greedy search metric to the ranking idea of these traditional methods.

#### 8.3.1. Kalman Filtering (KF)

KF, first introduced in [59], is an estimation method that uses a series of measurements observed over time and estimates the unknown variables within the system by estimating a joint probability distribution over the known variables for each time frame. It requires a dynamical system model represented by two equations: a state Equation (39) and an observation Equation (40).

(39) $$x_{k}=Fx_{k-1}+Bu_{k}+w_{k}$$

(40) $$z_{k}=Hx_{k}+v_{k}$$

In the above equations, *F* represents the state transition matrix, *H* represents the observation matrix, *B* is the control input model, $x_{k}$ is the state vector $z_{k}$ is the observation vector, $w_{k}$ is the system noise vector, $v_{k}$ is the observation noise vector, and $u_{k}$ is the control vector. The subscript *k* denotes the iteration number (or alternatively, time or sample).

We provide the steps of the linear KF process in Algorithm 5, where notation from the state and observation equations carry over, and $\hat{x}$ is the predicted estimation vector, $\hat{P}$ is the estimated covariance matrix, *R* is the covariance matrix of the measurement noise, and *Q* is the covariance matrix of the process noise.

If the system represented by Equations (39) and (40) are not linear or the noises are not Gaussian, the process seen in Algorithm 5 requires modifications, which is where the modified KF methods, such as EKF and UKF, emerge.
**Algorithm 5 **KF**Step 1:** InitializationInitialize the parameters of the state and observation models given in Equations (39) and (40) (initial states, observations, matrices, noises, etc.).**Step 2:** Predict State and Covariance   $x_{k}=F{\hat{x}}_{k-1}$   $P_{k}=F{\hat{P}}_{k-1}F^{T}+Q$**Step 3:** Compute Kalman Gain (*K*)   $S=HP_{k}H^{T}+R$   $K=P_{k}H^{T}+S^{-1}$**Step 4:** Correction   ${\hat{x}}_{k}=x_{k}+K\left(z-Hx_{k}\right)$   ${\hat{P}}_{k}=P_{k}+KHP_{k}$**Step 5:** TerminationRepeat steps 2–4 until a termination criterion is met (i.e., total iterations, change in optimality metric, etc.).

Both EKF and UKF apply a linearization step to *H* and/or *F* matrices before the equations in steps 2–4 of Algorithm 5 are applied to address this issue. In the case of EKF, the non-linear matrix *H* is approximated via a Taylor series expansion about the estimated state vector, as in Equation (41), where the notation from Equations (39) and (40) carries over.

(41) $$H\left[x_{k+1|k}\right]\approx H\left[x_{k|k}\right]+\frac{\partial H[x_{k|k}]}{\partial x_{k|k}}\left(x_{k+1|k}-x_{k|k}\right)$$

In the case of UKF, an unscented transformation is applied, as seen in Equation (42), where $\chi^{v}$ are the sigma points describing the measurement noise and $\chi^{x}$ are the sigma points describing the prior predicted states. $n_{x}$ is the number of weighted samples, which is chosen as a sum of the number of process states and the dimensions of $w_{k}$ and $v_{k}$. Again, notation carries over from Equations (39) and (40).

(42) $$H\left[x_{k+1|k}\right]\approx \sum_{i=0}^{2n_{\chi }}W_{i}\left[\chi_{i,k+1|k}^{x}\right]+\chi_{i,k+1}^{v}$$

While KF can be used as an offline or online algorithm depending on what the optimization objective is (i.e., what the system model from Equations (39) and (40) represents), in practical applications concerning autonomous vehicles, it is typically used in an online fashion. Although not always in the LEO context, the most common examples of KF implementations in the field of autonomous vehicles are sensor fusion [169] and/or trajectory tracking [170]. The method typically does not consume significant resources from the system and adds negligible delay to the operation; thus, it remains a highly utilized method, although not always for optimization but rather as a method for estimation, tracking, etc.

#### 8.3.2. Search Methods

Search methods are optimization calculations or algorithms that are employed to solve a search problem to varying degrees of optimality. There are a vast number of such methods, but we will do our best to give overviews for the commonly used ones with respect to the user segment problems seen in Table 2.

We start with some brute-force methods. Continuing with the satellite selection problem, an ’optimal’ brute-force method would be to look at all possible combinations of *k* out of *n* satellites to determine the best performance. Such brute-force methods are optimal in terms of returning the best possible outcome but are distinctly non-optimal in terms of overhead, i.e., computational cost and time. If there were *n* healthy satellites above the mask, a receiver with *k* channels would have to evaluate $N_{opt}$ geometries, where $N_{opt}$ is given by a simple combination, as shown in Equation (43). However, it is easy to see that the number of geometries to evaluate can quickly become infeasible as *n* and *k* change.

(43) $$N_{opt}=\frac{n!}{(n-k)!k!}$$

Another brute-force approach is to utilize a greedy heuristic to make the combination more feasible. This ’Greedy Search’ method is similar to the optimal, but the difference is that instead of calculating all possible combinations, the search focuses on the best subset of a chosen satellite, iterating over all the satellites. For an example, if we take an initial case with n=10 satellites, all subsets continuing 9 satellites are calculated first, and then the one with the best metric is selected to continue evaluating another subset of eight satellites. This results in an improved speed over the straightforward combination calculation, but it runs the risk of missing the global optimum. In addition, the overhead may still remain non-optimal.

More sophisticated search methods are typically employed to solve problems related to network routing optimization, which can be formulated as shortest path problems. The first algorithm we would like to introduce is the well-known ’Dijkstra’s Algorithm’, originally introduced in [46] to find the shortest path between two given nodes. Nowadays, the common variant finds the shortest path from a ’source’ node to all the other nodes in the network. We provide the steps in Algorithm 6.

This algorithm can be seen as a brute-search type of algorithm as it typically ends when the entire network is known (therefore, the overhead remains at the non-optimal levels). However, it can also be coupled with models depending on the available information about the network and how accurate its cost calculation and dynamic model is.

**Algorithm 6 **Dijkstra’s Algorithm
**Step 1:** InitializationGiven the network graph, assign a cost value to every node in the network; the cost value of a node is the total cost value of the shortest path discovered so far between that node and the source node. Initially, this means 0 for the source node and inf for all other nodes. Furthermore, create a list for unvisited nodes and initialize it with all the nodes in the network. Set the current node as the source node.**Step 2:** Calculate Cost to NeighborsFor the current node, consider all of its unvisited neighbors and calculate their cost values through the current node. Compare the newly calculated cost to the current assigned value and assign the smaller one.**Step 3:** Update Current NodeRemove the current node from the unvisited list and assign the neighbor with lowest cost as the current node that is in the unvisited list (a visited node will never be checked again).**Step 4:** TerminationRepeat steps 2–4 until a termination criterion is met (i.e., destination node is found, all routes in the network are known, etc.).


Another well-known algorithm is the ‘A* Search Algorithm’, which is a very similar algorithm to Dijkstra, except it includes a heuristic while selecting which nodes to explore next. As we have shown in Algorithm 6, Dijkstra decides which node to explore next based on the path cost. If we define the path cost from the current node to node *n* as g(n), then the decision function f(n) can be written as in Equation (44) for Algorithm 6.

(44) f(n)=g(n)

To employ the A* Search instead of Dijkstra, this decision function must be modified, as seen in Equation (45), where h(n) is some heuristic function, for example, a state value function, or a greedy function.

(45) f(n)=g(n)+h(n)

The general advantage of A* over Dijkstra is that as it uses a heuristic to select the nodes to explore, it is significantly faster than Dijkstra, which results in a more optimal overhead. However, as a result, its ability to find the global optimum is dependent on the heuristic function’s ability to represent the real network.

### 8.4. Method Comparisons for the User Segment

Finally, we compare the discussed optimization methods throughout this section in Table 11, similar to the way we did in Section 6 and Section 7. Note that the traditional and search methods entries are generalization terms that do not refer to the individual performance of the methods but to our interpretation of the overall performance of similar methods that utilize the same type of approach with varying success.

## 9. Design Recommendations

In this section, we compile our comparisons of the methods from Table 9, Table 10 and Table 11 in order to provide general recommendations for the optimization problems related to each LEO segment when having the application class of autonomous vehicles in mind. Our design recommendation can serve as a basis for the future design of LEO-based networks to meet the stringent requirements for autonomous transportation, as listed in Table 4.

Regarding the space segment, the current studies show that the best trade-off between various performance metrics can be achieved with the evolutionary algorithm variants (such as MOGA and MOPSO), as well as with ML-based approaches, such as constructing an DNN for the optimization problem, given a good dataset that represents the system high dynamics. However, specifically for the space segment, ML-based approaches seem to be a scarce case in the literature, which we attribute to the lack of available quality data regarding constellation optimization. As a result, we recommend focusing on the evolutionary algorithms, where the dominated methods are between GA and PSO variants that make use of smart modifications to guide the methods through the search space.

In terms of the ground-segment optimization, our literature review has shown that LSTM typically provides the best solution for the majority of applicable cases, followed by NSGA-II. Regarding the drawbacks of LSTM, as with most other data-driven approaches, its performance highly depends on the availability of a good training dataset that is able to reflect the main characteristics of the optimization problem at hand. In reality, such a quality dataset might not be easy or feasible to acquire. Therefore, our design recommendation for ground-segment optimization is to use NSGA-II optimization engines, providing a good tradeoff between different performance metrics. Indeed, in terms of performance, NSGA-II is not far behind LSTM, but since it does not depend on available training data, its application range is wider than the one of LSTM, and it is, in our opinion, more suitable for the application areas relying on stringent requirements, such as those from autonomous transportation. That being said, NSGA-II performance is heavily affected by the choice of the algorithm criteria, such as the probability models related to genetic operators, but fairly accurate models can be found in the literature and are relatively common.

As for the user segment, it is difficult to provide meaningful generic design recommendations, as this is the segment with the most amount of challenges and possible applications, as well as the segment most heavily affected by the nature of the above-mentioned problems or applications. We have provided different illustrative examples for characterizing the user-segment performance, e.g., based on $C/N_{0}$ or based on the average number of satellites in view by a terrestrial autonomous vehicle, and these can be used as a starting point for further exploration. In addition, the areas of applications from the user-segment point of view comprise communication, sensing, and navigation domains, with typically contradictory criteria to meet (e.g., wide receiver bandwidths and high carrier frequencies are highly suitable for high-throughput applications, while narrow bandwidths and smaller carrier frequencies are better for good link budgets and navigation applications in heavy urban or indoor scenarios).

There are two general notes we would like to add on top of our recommendations in the previous paragraphs. The first is about the modeling part; as it should be apparent from explanations in Section 6, Section 7 and Section 8, underlying theoretical models are needed and included in most steps of all methods, at the very least in the evaluation metrics. Thus, it is a logical conclusion that there will be models that will influence the results in an equal or higher manner than the influence of choosing a particular optimization method in a per case basis. However, creating generic models is again a challenging issue, especially in problems with conflicting goals, such as most multi-objective multi-modal problems, as the modeling parameters are typically hard to tune simultaneously in an optimal direction. This is one reason why optimization methods are widely utilized while still depending on partial or simplified models of the problems they are optimizing.

The second note is about ML-based methods, which include, for example, the NN and RL methods in Figure 3. It is important to remember that, despite their large-scale spread in the literature in all kinds of applications and problems, ML methods are still considered mostly ’black box’ models, which means that, while we are able to reliably explain ’what’ works in such models, we rarely have sufficient explanations to ’why’ or ’how’ they work, and the reproducibility of results cannot be automatically ensured. Due to this, they are very difficult to reproduce, which can lead to complications if applied in large-scale real-world applications and, in particular, for LEO system design for autonomous transportation applications.

## 10. Conclusions, Open Directions, and Future Studies

Our paper gave a broad overview of the optimization methods that can be used in the context of LEO system design for communication, navigation, and/or sensing applications within the future autonomous transportation applications. The optimization methods were presented both at a general level and segment-by-segment, following the three-segment architecture of most satellite systems (namely space, ground, and user segments). We have thoroughly identified the optimization metrics and constraints at each of the segments, and we compared various optimization methods in terms of their complexity, convergence characteristics, and feasibility. We have shown that the typical optimization issues in the LEO-system design context are complex, multi-objective, and multi-modal optimization problems, and therefore, they are to be tackled separately, segment-by-segment, with the target application criteria in mind. The targets in the context of future autonomous vehicles are stringent, as depicted in Table 4, and they can be reached with a combination of various complex optimization mechanisms, such as ML or PSO for the space segment, LSTM or NSGA-II for the ground segment, and a scenario-dependent optimization approach for the user segment. However, for the sake of higher control and reproduction of the results, it is our view that ML-based approaches are not the best-fit-to-problem in the context of LEO system design for autonomous vehicles, especially for safety-critical transportation applications, and therefore, methods such as PSO or NSGA-II are to be preferred.

In addition to the segment-specific optimization tasks addressed here, we have mentioned various other issues, such as space debris, standardization constraints, and security targets, which can be further incorporated as optimization boundaries, but which are too broad to be inserted in a single optimization problem.

While our paper has covered a broad area of LEO design aspects, issues such as physical-layer optimization (e.g., modulation, channel coding, and carrier frequency choice), medium access control (e.g., multi-access control scheme and joint multi-access-physical layer optimization), network routing aspects, or security aspects have not been addressed in detail and remain a matter of future investigation.

Furthermore, as future directions, there is currently a dichotomy of approaches regarding communication, sensing, and, especially, positioning applications with LEO signals: on one hand, one could use any of the existing systems (e.g., such as those listed in Table 7) as signals of opportunity or as a basis for offering novel services to the end users; on another hand, one could design completely new systems, such as the on-going efforts towards a LEO-PNT concept. In the former case, the burden of the optimization problems will stay mainly within the receiver (thus at the user-segment part) because the infrastructure is assumed to be already deployed; in the later case, a three-segment optimization is needed in a joint or disjoint (segment-by-segment) manner.

Interesting new research directions in this domain comprise the integration of LEO and MEO solutions, such as a GNSS receiver on-board LEO satellites to better support the PNT targets, integration of cellular (5G, Sixth-Generation of Cellular Networks (6G)) networks with LEO networks, the use of RL other ML approaches for increased security in the wireless communication and navigation links, beamforming-based positioning via ML, and LEO-based edge computing.

Our future work will focus on designing novel LEO-PNT constellations by applying the identified segment-by-segment optimization methods in order to achieve improved accuracy for indoor and dense-urban positioning of devices with global coverage.

## Figures and Tables

**Table 1:** Related Work in the Literature and Comparison with Our Survey.

Reference	Space Segment Optimization Aspects	Ground Segment Optimization Aspects	User Segment Optimization Aspects	LEO Networks	Autonomous Vehicles	Cost and/or Coverage Aspects
Guerra et al., 2018 [7]	❍	❍	❍	●	●	❍
del Portillo et al., 2018 [10]	❍	●	❍	●	❍	❍
Zolich et al., 2019 [11]	❍	❍	❍	◗	●	◗
Reid et al., 2020 [12]	◗	❍	❍	●	◗	◗
Guan et al., 2020 [13]	●	❍	❍	●	❍	❍
Hassan et al., 2020 [14]	◗	●	◗	◗	❍	◗
Papa et al., 2020 [15]	◗	❍	❍	●	❍	◗
Juan et al., 2020 [16]	❍	●	❍	◗	❍	❍
Tan et al., 2021 [17]	◗	◗	●	◗	❍	❍
Ma et al., 2021 [18]	◗	●	❍	❍	●	❍
Our survey	●	●	●	●	●	●

● = topic addressed in detail, ◗ = topic partially addressed, ❍ = topic not addressed.

**Table 2:** Examples of Optimization Problems per LEO Architectural Segment.

Optimization Segment	Optimization Problem	Example of Parameters	Common Metrics or Cost Functions	State-of-Art Methods
Space	Constellation Optimization	Keplerian Elements	$C/N_{0}$	GA [19]
		Altitude	Ground Coverage	PSO [20]
		Number and Density of Satellites	GDOP	SA [21]
		Number & Inclination of Orbital Planes	Production/Launch/ De-orbiting Costs	DNN [22]
		Phase Between Orbital Planes		Theoretical Modeling [23]
	Controller Placement	Number of Controller Nodes	Flow Setup Time Reconfiguration Time	Modeling [24]
		Controller Node Assignments	Latency	
Ground	GS Planning	Location of GS	Sky Coverage System Throughput	NSGA-II [25] LSTM [26]
		Number of GS	Link Capacity Deployment and Maintenance Costs	IGSD-MRM [27]
User	Satellite Selection	Number of Satellites	Antenna Elevation	Elevation Method [28]
		Satellite Geometry	GDOP	Heuristic Search [29]
		Number of Receiver Channels	Tracking Error	ML [30]
			Receiver complexity	Downdate Method [31]
	Orbit Estimation	Number of Satellites	RMSE	ML [32]
		Inclination	TDOP	
		Satellite Clock Bias	$C/N_{0}$	KF [33]
		Satellite Positions Satellite Velocities	SNR	
		Satellite Quaternions	SINR	Theoretical Modeling [34]
	Terrestrial vehicle or UAV Data Acquisition	Number of receiver sensors	Network Energy	Theoretical Modeling [35]
		Number of multipaths	Travel Time	
		Vehicle Location	BER	PSO [36]
		Vehicle speed	Transmitter-receiver distance	
		Vehicle Path		
	Network Routing	Number of Nodes	Ground Coverage	Brute-Force Methods [37]
		Link Assignment	Latency	
		Link Weights	Throughput	Heuristic Search [38]
		Number of Handovers	Handover Percentage	Joint Dynamic Optimization [39]
	Handover Planning	Handover Margin	Failure percentage	Lagrange Dual Method [40]
		Time-to-Trigger	SINR, SNR	
		Beam Selection	Number of Ping-Pongs	ML [41]
		Number of Rerouting Attempts	Latency	MARL [42]

**Table 3:** Types of Unconstrained Optimization Problems.

Type	Mathematical Formulations	Parameters
Single objective	$min_{x\in S}f\left(x\right)$	f(·) scalar, *x* scalar
Multi-objective	$min_{x\in S}\left(f_{1}\left(x\right),f_{2}\left(x\right),\ldots f_{N}\left(x\right)\right)$	$f_{i}\left(\cdot \right),i=1,\ldots ,N$ scalar, *x* scalar
Multi-modal	$min_{x\in S^{M}}f\left(x\right)$	f(·) scalar, $x={\left[x_{1},\ldots ,x_{M}\right]}^{T}$ vector
Multi-objective multi-modal	$min_{x\in S^{M}}\left(f_{1}\left(x\right),f_{2}\left(x\right),\ldots f_{N}\left(x\right)\right)$	f(·) scalar, $x={\left[x_{1},\ldots ,x_{M}\right]}^{T}$ vector

**Table 4:** Communications (C), Positioning (P), and Sensing (S) Requirements for Autonomous Vehicles.

Requirement	Domain	Ranges	Examples of Relevant References
High range for mobility support	C	0–1000 km/h	UAV airspeed estimation [67]; survey of sensor fusion techniques for all-speed autonomous vehicles [68]
High positioning accuracy	P, S	0.1–10 m	5G-based positioning [69]; positioning metrics in Cellular vehicle-to-everything (C-V2X) communications [70]; precision needed for fully autonomous driving [12]
High throughputs	C	0.1–50,000 Gbps	Air-to-ground (A2G) communications for flying vehicles [71]; high throughputs through cognitive internet of vehicles [72]
Low latencies	C, P, S	1–30 ms	LEO latencies compared with terrestrial network latencies [73]
High Coverage	C, P, S	>90%	Global coverage design [74,75]; CubeSat constellation design for IoT [76];
Environmental mapping	P, S	N/A	Aircraft runaway detection via remote sensing [77]; Space-Air-Ground integrated vehicular network [78]

**Table 5:** Main Parameters Describing LEO Constellations.

Constellation Parameters		Range	References
Altitude	*h*	200–2000 km	[88,89]
Number of Orbital Planes ${}^{1}$	$N_{P}$	≥4	[90]
Number of Satellites per Plane ${}^{1}$	$N_{S}$	≥7	[90]
Orbital Eccentricity	*e*	≈0	[91]
Orbital Inclination	*i*	65–$85^{\circ }$	[88]

^1^ These values correspond to a 1-fold coverage, namely the situation when each Earth point has at at least one satellite in view at a time.

**Table 6:** Walker and Flower Constellations Modeling Parameters.

Topology	Design Parameters	Common Parameters	Plane and Phase Separation
Walker Constellation	$i:N_{T}/N_{P}/F,h$	i,a	(2) $$\Delta \Omega_{jk}=2\pi \frac{\left(j-1\right)}{N_{P}}$$ (3) $$\Delta M_{jk}=2\pi \frac{N_{P}}{N_{T}}\left(k-1\right)+2\pi \frac{F}{N_{T}}\left(j-1\right)$$
Flower Constellation	$N_{p}$, $N_{d}$	i,a,e,ω	(4) $$\Delta \Omega_{k}=2\pi \frac{F_{n}}{F_{d}}\left(1-k\right)\;mod\left(2\pi \right)$$ (5) $$\Delta M_{k}=2\pi \frac{F_{n}N_{p}+F_{d}F_{h}\left(g\right)}{F_{d}N_{d}}\left(k-1\right)$$

**Table 7:** Examples of Main LEO Satellite Constellations with Corresponding Parameters.

Const.	# Sat	# $N_{P}$	*h* (km)	*i* (deg)	Band	Sat Mass (kg)	Application	Refs.
Globalstar	48	8	1414	45	S/L	700	Voice	[103,104]
Orbcomm	50	4	825	45	S	172	Voice	[105]
Iridium	66	6	780	86.5	K	689	Voice	[106]
Iridium NEXT	66	6	780	87	K	860	Broadband	[107]
SpaceX Starlink *	34,404	72/72/36 6/4/48 48/48/30 28/28/28 12/18	550/540 570/560 560/328 334/346 360/510 515/520 525/530 535/604 614	53/53.2 70/97.6 97.6/30 40/53 96.9/14 22/30 53/45 38/148 115.7	Ku/Ka/V	145	Broadband	[108,109]
OneWeb *	7808	18/12/8 36/32/32	1200	87.9/87.9 55/87.9 40/55	Ku/Ka	386	Broadband	[110,111]
Telesat *	1671	27/40	1015 1325	98.98 50.88	Ka	≈750	Broadband	[112]
Amazon Kuiper *	7774	28/36/34 28/36/34 652/325	590/610 630/590 610/630 640/650	33/42 51.9/33 42/51.9 72 80	Ka/V	N/A	Broadband	[113]
Xona Space *	≈300	N/A	≈800	N/A	C	N/A	Automotive domain	[8]
GeeSpace *	168	N/A	800/820	50/85	N/A	500	Automotive domain	[114]

* The parameters shown in the table are based on the latest information available, corresponding to the planned final constellation (status as of 5th of Feb 2022). Please check [8,108,110,112,113,114] for additional details.

**Table 8:** Launch Vehicles Classification.

Launch Vehicle LV	Payload Capacity to LEO [kg]
Micro-LV	≤500
Small-LV	501–2000
Medium-LV	2001–20,000
Heavy-LV	>20,000

**Table 9:** Comparative Summary of Optimization Methods for LEO Space-Segment Optimization.

Method	Convergence Speed	Global Convergence	Large-Scale Applicability
Theoretical Modeling	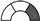	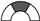	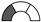
GA	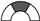	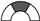	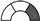
PSO	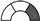	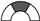	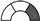
SA	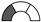	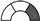	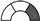
DNN	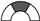	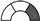	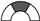

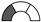
—Poor, 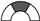
—Medium, 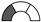
—Good.

**Table 10:** Comparative Summary of Optimization Methods for LEO Ground-Segment Optimization.

Method	Convergence Speed	Global Convergence	Large-Scale Applicability
Theoretical Modeling	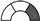	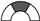	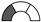
LSTM	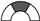	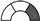	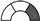
NSGA-II	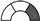	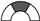	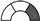
IGSD-MRM	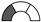	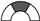	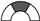

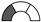
—Poor, 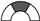
—Medium, 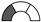
—Good.

**Table 11:** Comparative Summary of Optimization Methods for LEO User-Segment Optimization.

Method	Convergence Speed	Global Convergence	Large-Scale Applicability
Traditional Methods	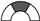	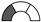	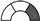
Search Methods	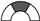	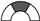	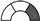
KF/UKF/EKF	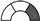	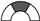	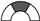
NN and DNN	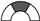	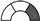	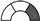
RL and MARL	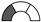	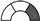	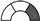

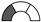
—Poor, 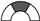
—Medium, 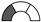
—Good.

## Acronyms

**3GPP**	Third-Generation Partnership Project
**5G**	Fifth-Generation of Cellular Networks
**6G**	Sixth-Generation of Cellular Networks
**AI**	Artificial Intelligence
**AKF**	Adaptive Kalman Filter
**AOL**	Argument Of Latitude
**BER**	Bit Error Rate
**CINR**	Carrier to Interference plus Noise Ratio
*C*/*N*_0_	Carrier to Interference plus Noise Ratio
**CNN**	Convolutional Neural Network
**DD**	Displacement Damage
Δ*V*	Impulse per unit of Mass (needed to perform a maneuver)
**DNN**	Deep Neural Network
**DOP**	Dilution of Precision
**DSSN**	Dense Small Satellite Network
**ECEF**	Earth-centered, Earth-fixed frame
**EKF**	Extended Kalman Filter
**EOL**	End-of-Life
**ESD**	Electro-Static Discharges
**FCC**	Federal Communications Commission
**GA**	Genetic Algorithm
**GDOP**	Geometric Dilution of Precision
**GEO**	Geo-Stationary Earth Orbit
**GNSS**	Global Navigation Satellite Systems
**GS**	Ground-Station
**HDOP**	Horizontal Dilution of Precision
**HRPSO**	Hybrid-Resampling Particle Swarm Optimization
**IGSD-MRM**	Iterative Ground Station Deployment based on Marginal Revenue Maximization
**INS**	Inertial Navigation Sensors
**IoT**	Internet of Things
**ITU**	International Telecommunication Union
**ITU-R**	International Telecommunication Union—Radio-communication unit
*J*_2_	Second-Degree Zonal Harmonic of the Earth’s Gravity Field
**KF**	Kalman Filtering
**LEO**	Low Earth Orbit
**LEO-NTN**	Low Earth Orbit-based Non-Terrestrial Network
**LEO-PNT**	Low Earth Orbit-based Positioning, Navigation, and Timing
**LOP**	Link Outage Probability
**LOS**	Line of Sight
**LSP**	Launch Service Provider
**LSTM**	Long Short-Term Memory
**LV**	Launch Vehicle
**MAC**	Multiple Access Channel
**MAE**	Mean Absolute Error
**MARL**	Multi-Agent Reinforcement Learning
**MC**	Monte-Carlo
**MEO**	Medium Earth Orbit
**MILP**	Mixed-Integer Linear Programming
**ML**	Machine Learning
**MOGA**	Multiple-Objective Genetic Algorithm
**MOPSO**	Multiple-Objective Particle Swarm Optimization
**MOSA**	Multiple-Objective Simulated Annealing
**NN**	Neural Network
**NSGA-II**	Non-Dominated Sorting Genetic Algorithm II
**PDOP**	Position Dilution of Precision
**PID**	Proportional-Integral Derivative
**PNT**	Position, Navigation, and Timing
**PSO**	Particle Swarm Optimization
**RAAN**	Right Ascension of the Ascending Node
**RL**	Reinforcement Learning
**RMSE**	Root Mean Square Error
**RNN**	Recurrent Neural Network
**SA**	Simulated Annealing
**SAGIN**	Space-Air-Ground Integrated Network
**SBAS**	Satellite-Based Augmentation System
**Sat**	Satellite
**SCIC**	Satellite Charging and Internal Charging
**SDN**	Software-Defined Networking
**SEE**	Single Event Effects
**SER**	Speech Emotion Recognition
**SINR**	Signal-to-Interference-plus-Noise Ratio
**SNR**	Signal-to-Noise Ratio
**SVM**	Support-Vector Machine
**TID**	Total Ionizing Dose
**TDOP**	Time Dilution of Precision
**UAV**	Unmanned Autonomous Vehicle
**UKF**	Unscented Kalman Filter
**VDOP**	Vertical Dilution of Precision
